# Manufacturing of non-viral protein nanocages for biotechnological and biomedical applications

**DOI:** 10.3389/fbioe.2023.1200729

**Published:** 2023-07-13

**Authors:** Jorge João, Duarte Miguel F. Prazeres

**Affiliations:** ^1^ iBB–Institute for Bioengineering and Biosciences, Department of Bioengineering, Instituto Superior Técnico, Universidade de Lisboa, Lisbon, Portugal; ^2^ Associate Laboratory i4HB–Institute for Health and Bioeconomy at Instituto Superior Técnico, Universidade de Lisboa, Lisbon, Portugal

**Keywords:** biomanufacturing, bottom-up synthesis, downstream processing, drug delivery, nanostructure engineering, protein nanocages, self-assembly, upstream processing

## Abstract

Protein nanocages are highly ordered nanometer scale architectures, which are typically formed by homo- or hetero-self-assembly of multiple monomers into symmetric structures of different size and shape. The intrinsic characteristics of protein nanocages make them very attractive and promising as a biological nanomaterial. These include, among others, a high surface/volume ratio, multi-functionality, ease to modify or manipulate genetically or chemically, high stability, mono-dispersity, and biocompatibility. Since the beginning of the investigation into protein nanocages, several applications were conceived in a variety of areas such as drug delivery, vaccine development, bioimaging, biomineralization, nanomaterial synthesis and biocatalysis. The ability to generate large amounts of pure and well-folded protein assemblies is one of the keys to transform nanocages into clinically valuable products and move biomedical applications forward. This calls for the development of more efficient biomanufacturing processes and for the setting up of analytical techniques adequate for the quality control and characterization of the biological function and structure of nanocages. This review concisely covers and overviews the progress made since the emergence of protein nanocages as a new, next-generation class of biologics. A brief outline of non-viral protein nanocages is followed by a presentation of their main applications in the areas of bioengineering, biotechnology, and biomedicine. Afterwards, we focus on a description of the current processes used in the manufacturing of protein nanocages with particular emphasis on the most relevant aspects of production and purification. The state-of-the-art on current characterization techniques is then described and future alternative or complementary approaches in development are also discussed. Finally, a critical analysis of the limitations and drawbacks of the current manufacturing strategies is presented, alongside with the identification of the major challenges and bottlenecks.

## 1 Introduction

In recent years, nanoparticles have been explored for applications in several scientific areas from nanobiotechnology and biomedical sciences to materials science and synthetic biology. Examples of nanoparticles studied in the literature include protein-based nanoparticles, metal nanoparticles, polymer micelles, silica nanoparticles and quantum dots (Lee, 2018). Among these, protein-based nanoparticles spurred significant research interest given their enormous potential for biomedical purposes (Schreiber and Schiller, 2013; Bhaskar and Lim, 2017; Lee, 2018).

In Nature, the existence of cellular processes essential to life such as metabolic reactions and information exchange is dependent on biological compartmentalization. In addition to lipids, proteins are one of the main components of natural compartmentalization systems such as virus capsids. Nanoparticles based on functional proteins constitute an additional example of such bio-compartments. Several of these protein-based nano-compartments, with different structural and functional characteristics, are described in the literature, including toroid- and donut-shaped proteins, tubes and yoctowells, protein nanocages, bacterial microcompartments (BMCs), protein membrane-based organelles (PMBOs) and gas vesicle protein nanoparticles (GVNPs) (Diekmann and Pereira-Leal, 2013; Lim, 2013; Schreiber and Schiller, 2013). For example, GVNPs, which have membranes exclusively composed of proteins, are produced in a wide variety of prokaryotic microorganisms, from heterotrophic bacteria to halophilic Archaea (e.g., *Halobacterium* sp. NRC-1). These nanostructures are spindle- or cylinder-shaped with a hydrophobic interior, having dimensions from 30 to 250 nm in width and from 50 nm to 2 µm in length. The most interesting properties of GVNPs include structural stability, monodispersibility, non-toxicity, self-adjuvanticity and ease of engineering. Some studies in the literature describe applications of GVNPs in drug delivery, in antigen display for vaccines, as contrast agents for ultrasound imaging and as acoustic biosensors (DasSarma and DasSarma, 2015; Andar et al., 2017; Hill and Salmond, 2020; Kim et al., 2022; Pfeifer, 2022; Karan et al., 2023). Nevertheless, among all these protein-based nanoparticles, protein nanocages are one of the most relevant (Diekmann and Pereira-Leal, 2013; Lim, 2013; Schreiber and Schiller, 2013).

Protein nanocages can be defined as highly ordered, nano-scale architectures. In general, they are produced through the self-assembly of multiple monomers, which may be identical or distinct, into symmetric and homogeneous structures of different shape and size. These protein-based nanoparticles allow spatial control of biological processes and compartmentalization of toxic, unstable, and sensitive compounds (Lee et al., 2016; Pieters et al., 2016; Diaz et al., 2018; Lee, 2018).

The advantages of protein nanocages result from their distinctive intrinsic characteristics, including a high surface/volume ratio, multi-functionality and ease of modification or manipulation through genetic or chemical strategies. Additionally, high stability, monodispersibility, biocompatibility, low toxicity and biodegradability are very attractive properties in the context of biotechnological and biomedical applications. Other applications include biomineralization and nanomaterial synthesis (Bhaskar and Lim, 2017; Diaz et al., 2018; Lee, 2018).

Protein nanocages can be classified as virus-like particles (VLPs) or non-viral protein nanocages (NVPNs). VLPs, which constitute the major group of protein nanocages, present an extensive variability in terms of structures and dimensions (Roldão et al., 2010; Patterson et al., 2012; Smith et al., 2012; Wen et al., 2012; Hassani-Mehraban et al., 2015; Fu and Li, 2016; Li et al., 2017). While structurally similar to viruses, VLPs are not infectious since they lack genetic material (Diaz et al., 2018). NVPNs, on the other hand, are unrelated to viral particles. NVPNs are formed by the self-assembly of protein monomers. This process is critically determined by the nature of the interface between adjoining subunits (Lim, 2013; Bhaskar and Lim, 2017). A wide variety of NVPNs with different structural and functional characteristics have been described in the literature.

Key biomedical applications of NVPNs include drug delivery, vaccine development and diagnostic bioimaging (Lai et al., 2009; Suci et al., 2010; Ren et al., 2012; Jeon et al., 2013; Toita et al., 2013; Zhen et al., 2013; Moon et al., 2014; Ra et al., 2014; Lee E. J. et al., 2015; Lee W.et al., 2015). Non-natural, bioinspired NVPNs can also be designed *de novo* through the assembly of artificial, functional monomers (Vázquez and Villaverde, 2010; Schreiber and Schiller, 2013; Bhaskar and Lim, 2017; Diaz et al., 2018).

The development of biomedical applications of NVPNs requires large amounts of pure and well-folded nanoassemblies (Theil, 2012). Consequently, more efficient, flexible, and universal bioprocess technologies are needed to transform NVPNs into clinically valuable products. The development of such biomanufacturing processes should be accompanied by the setting up of adequate quality control strategies to characterize both biological function and structure of the obtained nanocages (Lee et al., 2016).

This review first overviews general and relevant concepts related to NVPNs, followed by a presentation of their main applications in bioengineering, biotechnology, and biomedicine. The processes currently used to manufacture protein nanocages are described, and the most important aspects of upstream and downstream processing are highlighted. State-of-the-art characterization techniques are then presented and future alternative or complementary approaches in development are also discussed. Finally, a brief critical analysis of the drawbacks of the current manufacturing strategies is presented, alongside the identification of the major challenges.

## 2 Non-viral protein nanocages

### 2.1 General aspects

Non-viral protein nanocages are formed by multiple protein monomers that self-assemble into precisely defined, symmetric, homogeneous and complex structures (Lim, 2013; Molino and Wang, 2014; Pieters et al., 2016; Lee, 2018). These nanometer size (10–100 nm) particles may originate from different prokaryotes and eukaryotes. Their structural characteristics are critical for important cellular functions, which include storage of minerals, regulation of iron homeostasis, chaperone activity for the protection of other proteins in response to high temperature, protection of DNA from oxidative damage, cargo transport of nucleic acids and catalytic support for enzymatic reactions (Lim, 2013; Molino and Wang, 2014; Diaz et al., 2018).

### 2.2 Structural and functional characteristics

NVPNs are robust, monodisperse and water soluble, and present high biocompatibility and biodegradability (Lim, 2013; Corsi and Mazzucchelli, 2016; Bhaskar and Lim, 2017; Diaz et al., 2018). Furthermore, they can be chemically or genetically modified to extend functions and applications beyond the natural ones. Such strategies rely on molecular, genetic, and crystal structure information available in the literature (Uchida et al., 2007; Moon et al., 2014; Giessen, 2016; Diaz et al., 2018). This versatility and ability to be used as a multipurpose platform constitutes one of their most interesting features. For example, since most NVPNs have intrinsic catalytic characteristics as well as the ability to carry different molecules in the inner core, it is possible to optimize them as reaction vessels and templates to synthesize metallic nanoparticles (Lim, 2013).

New functionalities can be engineered at the interface between monomers, and the external and internal surfaces of protein nanocages (Lim, 2013; Bhaskar and Lim, 2017; Diaz et al., 2018; Chen et al., 2021). The external surface can be conjugated with functional ligands to improve targeting of a therapeutic cargo, cell penetration and biodistribution. These ligands can be peptides, epitopes, or other small molecules. The possibility for multiple conjugation with different ligands is also attractive and limited only by steric hindrance (Domingo et al., 2001; Kickhoefer et al., 2009; Choi et al., 2011; Kang et al., 2012; Lim, 2013; Murata et al., 2015). The amino acids in the monomers that compose the inner surface of the NVPNs can be replaced by specific amino acids to enable the creation of anchors for the loading of molecules with different dimensions. Depending on the type of nanocage and on the dimensions of the molecule to be encapsulated, this loading process and the subsequent binding to the inner surface can be mediated by chemical interactions (covalent, ionic, hydrophobic) or through protein-protein interactions.

### 2.3 Self-assembly mechanism

Self-assembly is the key to nanocage architecture. If the underlying mechanism is known (Zhang and Orner, 2011; Doyle et al., 2013; Lv et al., 2021), self-assembly can be modulated by destabilizing interactions at the subunit interface. Therefore, nanocage disassembly and reassembly can be induced, which allows controlling both the molecular cargo release from the inner core and the encapsulation of payloads (e.g., therapeutic molecules or enzymes). Several reports study the conditions that permit disassembly without irreversibly damaging the protein nanocage and the subsequent reassembly into its original architecture (Kim et al., 2011; Lim, 2013; Ferrer-Miralles et al., 2015). Some of the most relevant factors are the pH (Dalmau et al., 2009; Kim et al., 2011; Peng and Lim, 2011), the ionic strength (Sánchez-Sánchez et al., 2014), the presence of reducing agents (Shen et al., 2015) and the presence of metals (Swift et al., 2009; Belval et al., 2016).

### 2.4 Designed NVPNs

Artificial nanocages can be designed and generated *de novo* by mimicking the intrinsic mechanisms of self-assembly of natural NVPNs. Starting from the structural characteristics (e.g., geometry, size) required for the target nanocage, functional monomers are selected and modified accordingly. The amino acid sequences in each monomer can be partially derived from natural nanocages or designed anew to promote self-assembly. Strategies and sub-methodologies used may include directed evolution, use of fusion proteins, redesign of key interfaces and the *de novo* design (Lv et al., 2021). Ultimately, monomer selection and design must guarantee that protein-protein interactions take place with minimal nonspecific aggregation (Vázquez and Villaverde, 2010; Lai et al., 2012b; Lim, 2013; Bhaskar and Lim, 2017; Diaz et al., 2018; Chen et al., 2021).

Hybrid protein nanocages, which are conjugated with components such as polymers, nucleotides, carbon hydrates or lipids, are yet another group of protein nanocages that can be considered. These nanocages may be of major importance for diagnosis and therapy applications, for example, in the context of targeting or modulation of immune response. However, hybrid NVPNs are not extensively described in the literature (Bhaskar and Lim, 2017).

### 2.5 Functionalization

Natural and artificial NVPNs can be functionalized to create nanoarchitectures more adjusted to the end applications (Moon et al., 2014; Giessen, 2016; Bhaskar and Lim, 2017). Two main strategies for the functionalization of NVPNs are described: genetic engineering and bioconjugation (Diaz et al., 2018). Genetic modifications allow a more precise control over the number, position and distribution of the introduced molecules (Lee et al., 2016). Specific techniques used include modular assembly (Kang et al., 2012; Guimaraes et al., 2013), improvement of payload encapsulation (Seebeck et al., 2006; Kar et al., 2011; Tamura et al., 2015), interface engineering (Peng et al., 2015; Chen et al., 2016), peptide display (Laplagne et al., 2004; Flenniken et al., 2006), and protein display (Phippen et al., 2016). Bioconjugation on the other hand consists in the attachment of molecules that cannot be introduced through genetic engineering. The conjugation can be performed through covalent (Flenniken et al., 2006; Falvo et al., 2013) or non-covalent bonds (Bhaskar and Lim, 2017; Diaz et al., 2018) (Bhaskar and Lim, 2017). The available literature indicates that genetic modification alone or in combination with bioconjugation is clearly the most efficient approach to modify NVPNs (Diaz et al., 2018).

### 2.6 Examples of natural and artificial NVPNs

**FIGURE 1:**
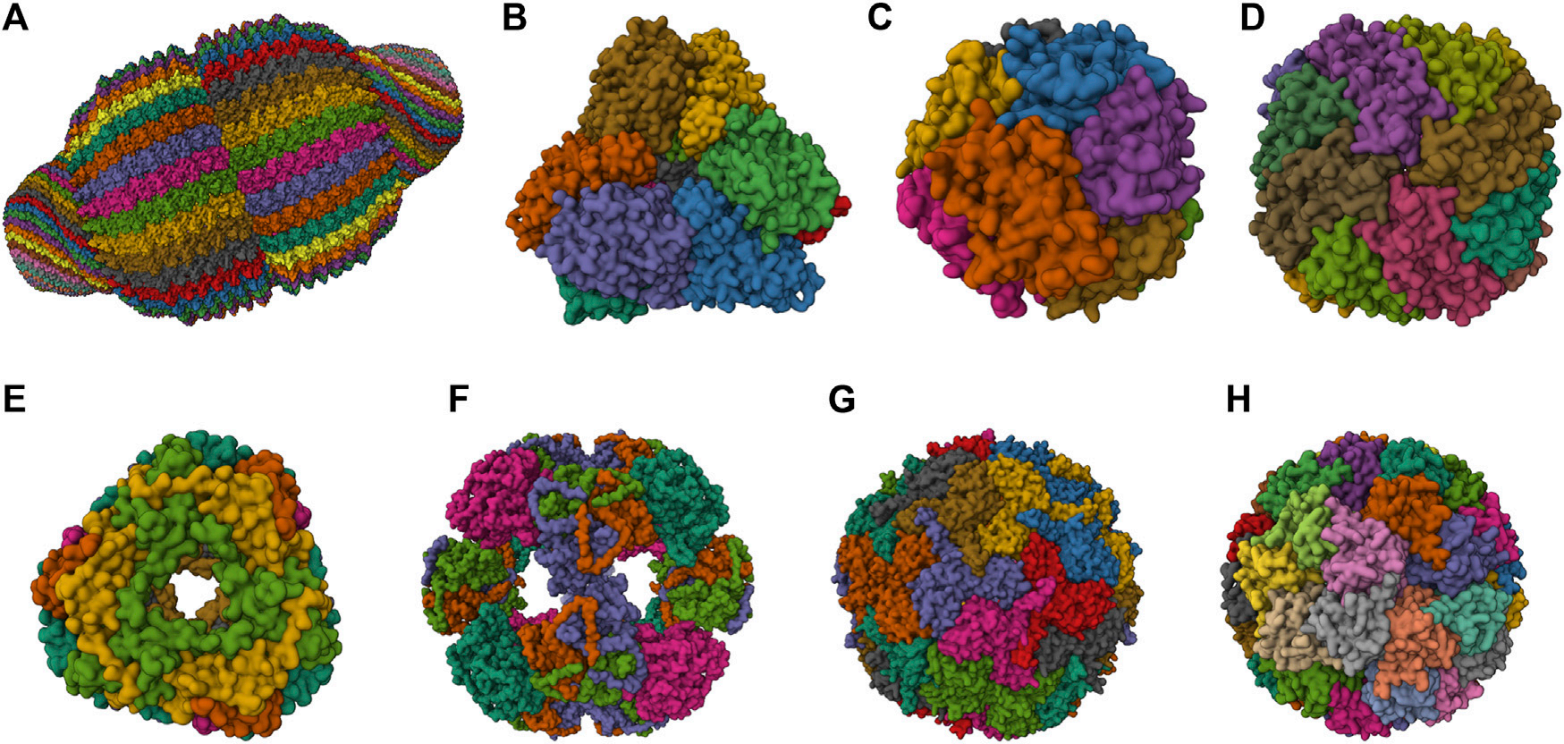
3D structures of the most studied natural NVPNs. **(A)** Vault (PDB ID: 4V60) (Tanaka et al., 2009). **(B)** Aminopeptidase (PepA) (PDB ID: 3KL9) (Kim et al., 2010). **(C)** DNA-binding protein from starved cells (Dps) (PDB ID: 1QGH) (Ilari et al., 2000). **(D)** Ferritin (PDB ID: 2FHA) (Hempstead et al., 1997). **(E)** Heat shock protein (HSP) (PDB ID: 1SHS) (Kim et al., 1998a). **(F)** Dihydrolipoyl acetyltransferase (E2) (PDB ID: 1B5S) (Izard et al., 1999). **(G)** Encapsulin (PDB ID: 3DKT) (Sutter et al., 2008). **(H)** Lumazine synthase (PDB ID: 1RVV) (Ritsert et al., 1995). Representations created using the Mol* Viewer tool (Berman et al., 2000; Sehnal et al., 2021).

**FIGURE 2:**
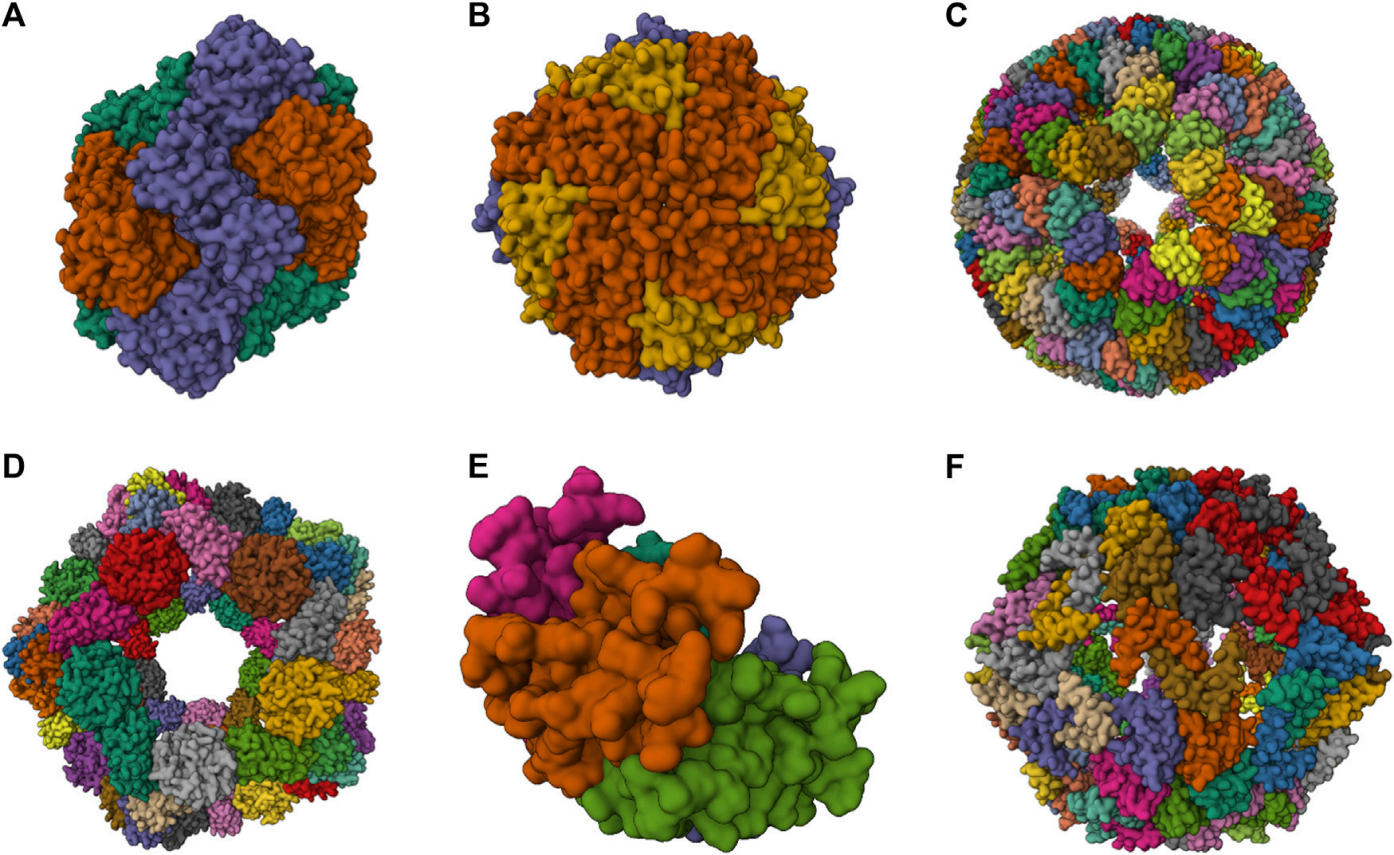
3D structures of some artificial NVPNs. **(A)** 16-nm protein nanocages designed (PDB ID: 3VDX) (Lai et al., 2012a). **(B)** His6-HuHF and His6-SF nanocages (PDB ID: 7CPC) (Gu et al., 2020). **(C)** trp RNA-binding attenuation protein (TRAP) nanocages (PDB ID: 6RVW) (Malay et al., 2019). **(D)** I3-01 nanocages (PDB ID: 8ED3) (McCarthy and Gonen, 2022). **(E)** Prototype oxygen-impermeable protein nanocages (OIPNC) (PDB ID: 7WKC) (Gao et al., 2022). **(F)** TIP60 nanocages (PDB ID: 7EQ9) (Obata et al., 2021). Representations created using the Mol* Viewer tool (Berman et al., 2000; Sehnal et al., 2021).

A broad spectrum of NVPNs, including both natural and artificial variants, are documented in literature. Figure 1 presents a schematic representation of the three-dimensional (3D) structure of natural NVPNs that have been extensively studied. Similarly, Figure 2 shows a schematic representation of the 3D structure of some artificial NVPNs.

#### 2.6.1 Natural NVPNs

The most relevant structural and functional characteristics of representative natural NVPNs are summarized in Table 1. Vault nanocages (Figure 1A) have extending caps at their ends, a hinged waist region, 8 small pores (2 nm in diameter) and a significant internal volume. Each vault nanocage is composed of several protein and non-protein elements, with the major vault protein (MJV) representing 70% of the overall mass. An interesting property is the dynamic ability of the vault nanocages, since they can open and close transiently, allowing the incorporation of small molecules and macromolecules within the inner core (Kickhoefer et al., 1996; Kickhoefer et al., 2005; Kong et al., 1999; Poderycki et al., 2006; Anderson et al., 2007; Ryu et al., 2008; Goldsmith et al., 2009; Tanaka et al., 2009; Yu et al., 2017).

**TABLE 1:** A summary of the structural and functional characteristics of the most studied natural NVPNs. The indicated code corresponds to the respective PDB ID.

Protein nanocages	Structural characteristics				Functional characteristics		References
	Number of monomers	Molecular weight	Geometry	Dimensions^a^	Native organism	Biological function	
Vault (4V60, Figure 1A)	78–96	13 MDa	Dihedral (39-fold)–Barrel-like structure	OL: 75 nm; OW: 42 nm	Eukarya (Example: *Rattus norvegicus*)	Involved in intracellular transport, cell signalling, cell survival and innate immunity	Kong et al. (1999), Poderycki et al. (2006), Anderson et al. (2007), Tanaka et al. (2009)
Aminopeptidase (PepA) (3KL9, Figure 1B)	12	457 kDa	Tetrahedral	OD: 12 nm; ID: 6 nm	*Streptococcus pneumonia*	Hydrolysis of oligopeptides into free amino acids	Kim et al. (2010), San et al. (2011), SIB Swiss Institute of Bioinformatics (2023)
DNA-binding protein from starved cells (Dps) (1QGH, Figure 1C)	12	216 kDa	Tetrahedral	OD: 9 nm; ID: 5 nm	*Listeria innocua*	Prevention of oxidative damage of DNA	Ilari et al. (2000), Kang et al. (2008), Haikarainen and Papageorgiou (2010)
Ferritin (2FHA, Figure 1D)	24	509 kDa	Octahedral	OD: 12 nm; ID: 8 nm	*Homo sapiens*	Regulation of the storage and release of iron	Zhang and Orner (2011), He and Marles-Wright (2015), SIB Swiss Institute of Bioinformatics (2023)
Heat shock protein (HSP) (1SHS, Figure 1E)	24	396 kDa	Octahedral	OD: 12 nm; ID: 6.5 nm	*Methanococcus jannaschii*	Chaperone activity in response to cellular stress	Kim et al. (1998a), Kim R. et al. (1998), Kim et al. (2003), Bova et al. (2002), Flenniken et al. (2003)
Dihydrolipoyl acetyltransferase (E2) (1B5S, Figure 1F)	60	1.6 MDa	Icosahedral	OD: 24 nm; ID: n.a	*Bacillus stearothermophilus*	Component of the pyruvate dehydrogenase (PDH) multienzyme complex	Domingo et al. (2001), Milne et al. (2006), Dalmau et al. (2008), SIB Swiss Institute of Bioinformatics (2023)
Encapsulin (3DKT, Figure 1G)	60	1.9 MDa	Icosahedral	OD: 24 nm; ID: 20 nm	*Thermotoga maritima*	Involved indirectly in oxidative stress responses through the encapsulation of other related proteins	Sutter et al. (2008), Tamura et al. (2015), Cassidy-Amstutz et al. (2016), Giessen (2016)
Lumazine synthase (1RVV or 1HQK, Figure 1H)	60	1 MDa	Icosahedral	OD: 14.7 nm; ID: 7.8 nm	*Bacillus subtilis, Aquifex aeolicus*	Enzyme complex involved in the synthesis of lumazine (riboflavin precursor)	Ritsert et al. (1995), Zhang et al. (2001), Ladenstein et al. (2013)

^a^ OL, outer length; OW, outer width; OD, outer diameter; ID, inner diameter; n.a, not available.

PepA nanocages are formed by the self-assembly of aminopeptidase (PepA), a zinc-dependent metallopeptidase (Figure 1B). The resulting nanoparticles have an inner cavity with negative charge and 8 pores on the edges and faces with diameters of 1 and 3 nm. The channels are useful for the traffic of molecules. PepA nanocages were used as templates for the size-controlled synthesis of ultrasmall platinum nanoparticles, with the formation of a multifunctional biohybrid catalyst (Kim et al., 2010; San et al., 2011).

DNA-binding protein from starved cells (Dps) belongs to the ferritin subfamily. Dps nanocages (Figure 1C) have small pores (0.8 nm) through which small molecules can diffuse. Dps nanocages were used as templates for the formation of metallic-protein nanoparticles to improve the endogenous catalytic activity of ferritins (Ilari et al., 2000; Kang et al., 2008; Haikarainen and Papageorgiou, 2010).

Ferritin nanocages (Figure 1D) have an internal cavity that matches the size of the iron core. In this core, iron is stored in an insoluble non-toxic state. The cages have 8 hydrophilic pores (4 nm) that facilitate the movement of iron atoms and other small molecules. Ferritin nanocages are quite stable at high temperature and in a wide range of pH values. Self-assembly, disassembly and reassembly can be controlled by metal ions. The nanocages were applied in biomineralization and as nanocarrier of biological and non-biological molecules (Lawson et al., 1991; Hempstead et al., 1997; Zhang and Orner, 2011; He and Marles-Wright, 2015; Kuruppu et al., 2015; Chen et al., 2016; Wang et al., 2017; Palombarini et al., 2020; Zhang et al., 2020).

Small heat shock protein (sHSP) nanocages (Figure 1E) have 8 pores (3 nm and 17 nm) that allow molecular trafficking. Advantages of the cages include high stability at high temperatures (up to 70°C) and in a broad range of pH values (5–11). sHSP nanocages were used as nanoreactors, for biomineralization, for drug delivery and for bioimaging. The functionalization reported includes the introduction of modifications with organic molecules outside and inside of the nanocages (Kim et al., 1998a; Kim et al., 1998b; Kim R. et al., 1998; Bova et al., 2002; Flenniken et al., 2003; Kim et al., 2003; Varpness et al., 2005; Flenniken et al., 2006; Abedin et al., 2009).

Dihydrolipoyl acetyltransferase (E2) nanocages from *Bacillus stearothermophilus* (Figure 1F) have 12 pores (5 nm) and high stability at extreme temperatures due to the thermophilic nature of the native organism. E2 proteins can be modified simultaneously at the outer and inner surfaces to allow loading of drugs inside and to display functional epitopes outside (Izard et al., 1999; Domingo et al., 2001; Jung et al., 2002; Milne et al., 2006; Dalmau et al., 2008).

Encapsulin nanocages (Figure 1G) have a characteristic large central cavity (20 nm) that makes them interesting as a cargo delivery nanoplatform and as a nanoreactor (Sutter et al., 2008; Rahmanpour and Bugg, 2013; Moon et al., 2014; Tamura et al., 2015; Cassidy-Amstutz et al., 2016; Giessen, 2016). Lumazine synthase nanocages (Figure 1H), which have a 7.8 nm inner cavity with negative charge, were used as molecular carriers through encapsulation. Other applications include the biomineralization of iron. A key characteristic of these nanocages is the stability at high temperatures (up to 95°C) (Schott et al., 1990; Ritsert et al., 1995; Shenton et al., 2001; Zhang et al., 2001; Seebeck et al., 2006; Ladenstein et al., 2013; Ra et al., 2014).

#### 2.6.2 Artificial NVPNs

Table 2 summarizes structural characteristics of artificial NVPNs reported in the literature. The formation of symmetric and homogenous 16-nm protein nanocages (Figure 2A) results from the self-assembly of monomers obtained by a fusion process. Each monomer is a geometrically controlled fusion of two natural protein oligomers, which are connected by an α-helical linker. One of those oligomers is the trimeric bromoperoxidase and the other is a dimeric M1 virus matrix protein (Lai et al., 2012a).

**TABLE 2:** A summary of the structural characteristics for some artificial NVPNs. The indicated code corresponds to the respective PDB ID.

Protein nanocages	Structural characteristics				References
	Number of monomers	Molecular weight	Geometry	Dimensions^a^	
16-nm protein nanocages designed (3VDX, Figure 2A)	12	600 kDa	Tetrahedral	OD: 16 nm; ID: n.a	Lai et al. (2012a)
His6-HuHF and His6-SF nanocages (7CPC, Figure 2B)	24	514 kDa	Octahedral	OD: 12 nm; ID: n.a	Gu et al. (2020)
trp RNA-binding attenuation protein (TRAP) nanocages (6RVW, Figure 2C)	24	2.2 MDa	Octahedral	OD: 22 nm; ID: 16 nm	Heddle et al. (2006), Heddle et al. (2007), Malay et al. (2019), Naskalska et al. (2021), Majsterkiewicz et al. (2022), Stupka et al. (2022)
I3-01 nanocages (8ED3, Figure 2D)	60	1.3 MDa	Icosahedral	OD: 26 nm; ID: n.a	Hsia et al. (2016), Votteler et al. (2016), Bruun et al. (2018), McCarthy and Gonen (2022), SIB Swiss Institute of Bioinformatics (2023)
Prototype oxygen-impermeable protein nanocages (OIPNC) (7WKC, Figure 2E)	n.a	n.a	Icosahedral	OD: 14 nm; ID: n.a	Gao et al. (2022)
TIP60 nanocages (7EQ9; Figure 2F)	60	1.1 MDa	Icosahedral	OD: 21.7–24.7 nm; ID: 15 nm	Kawakami et al. (2018), Obata et al. (2021), Ohara et al. (2023)

^a^ OD, outer diameter; ID, inner diameter; n.a, not available.

His6-HuHF and His6-SF nanocages (Figure 2B) with regulatable self-assembly were created based on two recombinant ferritins (rHuHF and rSF). Histidine motifs were incorporated in one of their subunit interfaces. Two different switches (metal- and pH-based) were developed to control the assembly-disassembly of the nanocages. This may lead to more efficient encapsulation of molecules within the nanocages compared to the traditional methods reported (Gu et al., 2020).

The trp RNA-binding attenuation protein (TRAP) nanocages (Figure 2C) result from the assembly of 24 ring-shaped proteins derived from the natural TRAP from *B. stearothermophilus*. Each constituent TRAP ring is formed by 11 monomers that were engineered to include a cysteine residue. Contrary to the usual situation, the complete nanocage is formed not through a network of protein-protein interactions but through the bridging of opposing thiols of the cysteine residues between TRAP rings via single gold (I) ions. The fully assembled TRAP nanocages present six square apertures (4 nm). TRAP nanocages are stable up to 95°C and at high concentration of denaturing agents (e.g., 7 M urea). Although they are susceptible to reducing agents, this could be a promising characteristic for delivery to targets that contain this type of agents. The fact that the assembly relies on a metal-induced process is very useful since it provides a more rigorous control of assembly-disassembly and, eventually, a programmable mechanism. The nanocages can also be labelled with a dye in each ring-shaped monomer, which could be useful for bioimaging applications (Heddle et al., 2006; Heddle et al., 2007; Malay et al., 2019; Naskalska et al., 2021; Majsterkiewicz et al., 2022; Stupka et al., 2022).

I3-01 nanocages (Figure 2D) are hollow architectures that result from the self-assembly of multiple monomers corresponding to a trimeric 2-keto-3-deoxy-6-phosphogluconate (KDPG) aldolase from *Thermotoga maritima*. These aldolase monomers were engineered to contain complementary hydrophobic interfaces. The nanocages have several large pores (9 nm). I3-01 nanocages are stable up to 80°C and in the presence of high concentration of denaturing agents (e.g., 6.7 M guanidine hydrochloride). Applications include synthetic biology, targeted drug delivery, and vaccine development (Hsia et al., 2016; Votteler et al., 2016; Bruun et al., 2018; McCarthy and Gonen, 2022).

Prototype oxygen-impermeable protein nanocages (OIPNC) (Figure 2E) are derived from the pentameric β-carboxysome from *Thermosynechococcus elongatus* BP-1 (CcmL). Self-assembly of the proteins into nanocages occurs in the presence of quantum dots as templates by protein-quantum dots interfacial engineering. An advantage of these nanocages is the permeability to O_2_ in a switchable process controlled by molecular patches. Future interesting applications include their use as nanocarriers and nanoreactors (Gao et al., 2022).

TIP60 nanocages (Figure 2F) are hollow spheres with 20 triangular pores that were created using a fusion protein design approach. Each monomer is a genetic fusion of two proteins (one pentameric- LSm and one dimeric- MyoX-coil) with a three-residue linker. A reversible assembly and disassembly mechanism based on metal ions and chelators was developed for TIP60 nanocages (Kawakami et al., 2018; Obata et al., 2021; Ohara et al., 2023).

## 3 Applications of non-viral protein nanocages

Applications of NVPNs can be found in a wide variety of areas and fields of study, with particular emphasis on those related to bioengineering, biotechnology, and biomedicine. Representative applications in drug delivery, vaccine development, bioimaging and diagnostic imaging, biomineralization and nanomaterials synthesis, and biocatalysis are briefly discussed in the following sections and presented with more details in the Supplementary Table S1.

### 3.1 Drug delivery

NVPNs constitute an excellent vehicle for the encapsulation, targeted delivery, and controlled release of drugs, which can range from small molecules to larger biomolecules like nucleic acids or proteins. Several NVPNs (e.g., Dps, encapsulin, ferritin, sHSP, and TRAP nanocages) were functionalized through genetic or chemical modifications to contain targeting molecules (e.g., biotin, hepatocellular carcinoma cell binding peptides, neuropilin 1-binding peptides and PTD4 cell-penetrating peptides) and to allow the incorporation of different cargos (e.g., SnCe6 photosensitizer, aldoxorubicin, doxorubicin, OSU03012 anticancer drug, small interfering RNA, curcumin). In general, results show that NVPN-mediated delivery can be performed successfully and that the desired effect is achieved efficiently (Suci et al., 2010; Bellini et al., 2014; Moon et al., 2014; Murata et al., 2015; Guan et al., 2018; Naskalska et al., 2021; Ji et al., 2022).

### 3.2 Vaccine development

NVPNs can be used as platforms for antigen display, offering the possibility of co-delivery of adjuvants, targeted delivery, immune modulation, and antigen stabilisation. Engineered protein nanocages including E2, ferritin, I3-01, lumazine synthase, sHSP and vault nanocages were demonstrated as a potential vaccine platform, with the triggering of strong immune responses (namely, CD8^+^ and CD4^+^ T-cell responses). Different types of antigens and other molecules were displayed on the outer and/or inner surfaces of the nanocages, such as human melanoma-associated antigen gp100, MHC I-restricted SIINFEKL peptide epitopes, SIINFEKL and ISQAVHAAHAEINEAGR peptides, and transmission-blocking and blood-stage malaria antigens (Wang et al., 2003; Kar et al., 2012; Molino et al., 2013; Han et al., 2014; Ra et al., 2014; Bruun et al., 2018).

### 3.3 Bioimaging and diagnostic imaging

The incorporation of contrast agents into NVPNs offers the possibility of extending their applications to the visualization of biological processes, detection of diseases, and monitoring of therapies. Ferritin and sHSP nanocages were tested in the context of bioimaging and diagnostic imaging applications. For example, the conjugation of NVPNs with targeting peptides (e.g., RGD and DEVD) and a fluorescent molecule (e.g., Cy5.5) allowed the imaging of caspase activity inside tumor cells (Choi et al., 2011). In another study, labelled protein nanocages loaded with an iron oxide nanoparticle catalyzed the oxidation of peroxidase substrates, which allowed the subsequent visualization of tumor tissue (Fan et al., 2012). Protein nanocages combined with metallic nanoparticles also showed promise in real-time *in vivo* photoacoustic imaging of tumor cells, and in positron emission tomography imaging when combined with a copper radionuclide (Wang et al., 2016). Finally, molecular magnetic resonance imaging was possible with a protein nanocage engineered with a targeting molecule (e.g., neuropilin 1-binding peptide) and combined with gadolinium (III)-chelated contrast agents (Kawano et al., 2018).

### 3.4 Biomineralization and nanomaterials synthesis

NVPNs can be used to control and direct the formation of nanomaterials with specific properties, for example, by serving as templates for the growth of inorganic minerals, by encapsulating metal nanoparticles, quantum dots, or magnetic nanoparticles, or by acting as microreactors for the controlled synthesis of nanomaterials. For example, natural and engineered Dps and sHSP nanocages were used as a nanoscale platform for the synthesis of monodisperse and homogeneous iron oxide nanoparticles (Allen et al., 2002; Flenniken et al., 2003). In another application, a synthetic polymer with modifiable groups was successfully incorporated into HspG41C protein nanocages (Abedin et al., 2009).

### 3.5 Biocatalysis

The use of NVPNs for enzyme encapsulation and immobilization, substrate channelling and modulation/tuning of enzyme properties offers an opportunity to improve catalytic efficiency, alter selectivity and specificity, and enhance stability and recyclability. For example, bioinorganic hybrid catalysts with interesting characteristics (namely, greater stability and prevention of agglomeration) were created by incorporating an enzyme (e.g., manganese peroxidase) inside a NVPN (such as ferritin, PepA, sHSP and vault nanocages) and using the biomineralization capacities (e.g., iron oxide and platinum) of these nanostructures (Ensign et al., 2004; Varpness et al., 2005).

## 4 Non-viral protein nanocages manufacturing

### 4.1 Overview

Efficient manufacturing of NVPNs nanostructures, all the way from the laboratory to the industrial scale, is crucial for the development of applications. However, few scientific studies available in the literature have dealt with the biomanufacturing of natural and artificial NVPNs. In addition to basic and applied research, it is essential to focus on bioprocess development, as this will play a pivotal role in bringing protein nanocages closer to the market.

**FIGURE 3:**
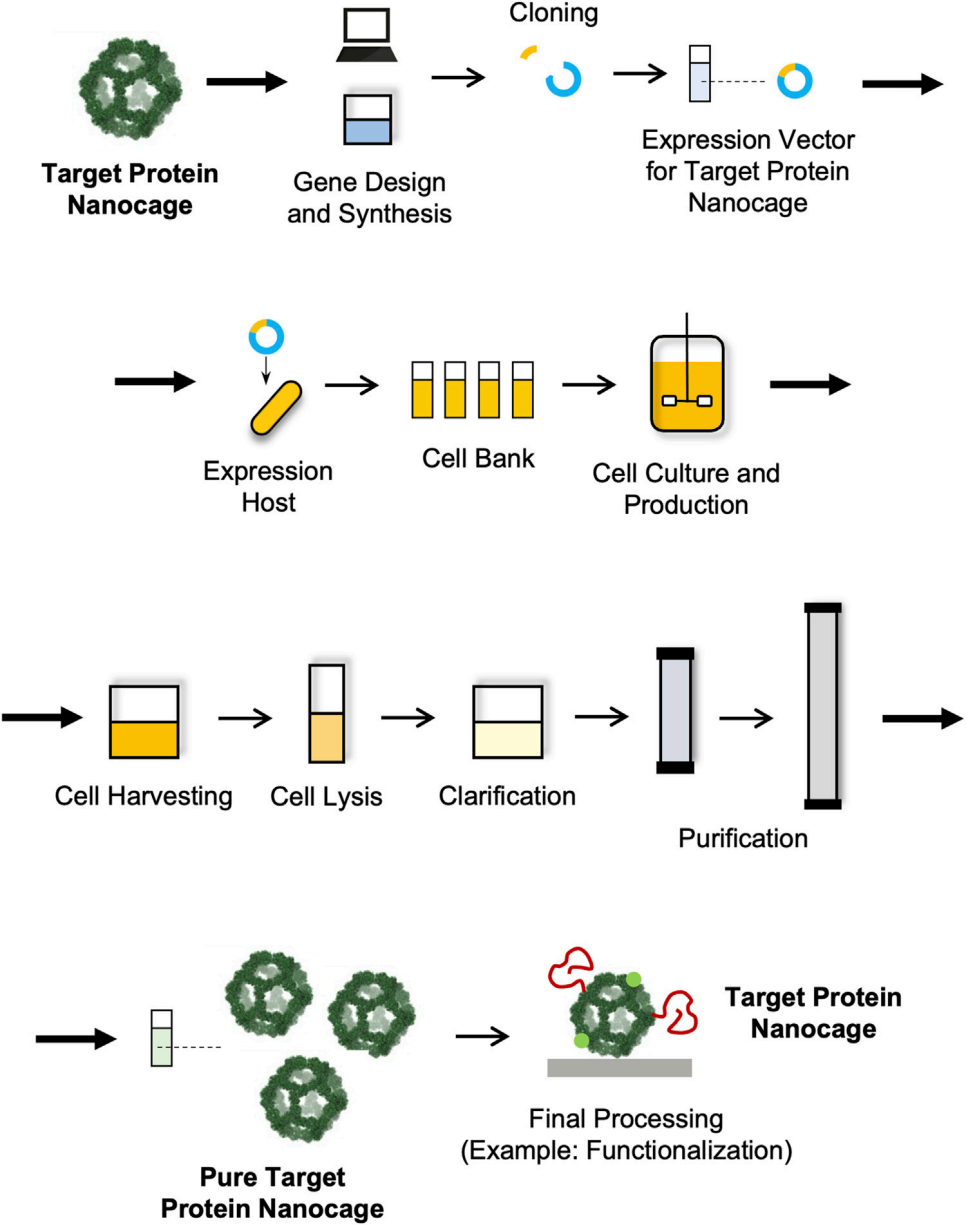
Schematic representation of a manufacturing process for NVPNs.

Like in the case of other biological and biopharmaceutical products, the manufacturing of NVPNs involves a sequence of actions that are designed with the objective of producing a certain amount of product with specific quality features (João et al., 2021). These actions can be categorized into the upstream and the downstream processing sections (Section 4.2 Upstream Processing and Section 4.3 Downstream Processing) (Figure 3). The upstream processing involves the generation of the producer host cells (including host selection and cloning), cell banks implementation, inoculum preparation, cell cultivation and protein nanocage expression (Owczarek et al., 2019; Puetz and Wurm, 2019). The downstream processing includes all the unit operations required to purify the NVPNs to a point where final product specifications are met. Lastly, the manufacturing will require a final processing stage, which may comprise formulation, functionalization and sterilization steps to yield the desired final nanocages (Diaz et al., 2018; João et al., 2021).

Since NVPNs are complex biologicals with an intrinsic variability in terms of composition, stability and biological activity, another important aspect to consider during process development is the implementation of analytical and characterization approaches (Section 4.4 Analytical and Characterization Technologies). Clearly, the set-up of such methodologies is essential to determine and evaluate the structural and functional features of the final nanocages, but also to adequately monitor the performance of the biomanufacturing process throughout all its steps (João et al., 2021).

### 4.2 Upstream processing

Cultivation of producer host cells and protein expression is at the core of a NVPN manufacturing process. While the ultimate purpose is to produce large amounts of protein nanocages, scientific studies focused on application development require amounts that can be generated easily with simple lab-based protocols. In general, these protocols are directed at low-volume batches and do not rely on a strict control of operating conditions. This results in low host cell densities and in reduced nanocage yields. New and optimized strategies are clearly required that can be used in large-scale settings in order to increase volumetric productivity and consequently decrease production costs (Diaz et al., 2018; Tripathi and Shrivastava, 2019; Chen et al., 2021; João et al., 2021).

#### 4.2.1 Selection of host cells

The upstream processing comprises the selection of the host cells, the preparation and optimization of the expression vector and its subsequent transformation/transfection into the host. All these aspects are crucial for obtaining a quality bioproduct and a high productivity (Puetz and Wurm, 2019; Tripathi and Shrivastava, 2019). While the natural host can be selected if the desired NVPN is expressed naturally (Schott et al., 1990; Sutter et al., 2008), nanocage production uses mainly expression systems/host dedicated to recombinant protein production (Diaz et al., 2018). Here, a variety of expression hosts are available that include bacteria, yeasts and multicellular fungi, insect, mammalian and plant cells (Diaz et al., 2018; Puetz and Wurm, 2019; Tripathi and Shrivastava, 2019). NVPNs are mostly produced recombinantly in bacterial cells, and in particular in *Escherichia coli*, a very-well studied host that grows fast, is easy to cultivate and propagate at low cost, and displays high productivity. However, *E. coli* is also associated with disadvantages such as the lack of proper post-translational modifications (PTMs), the formation of inclusion bodies and the propensity to generate endotoxin contamination due to its Gram-negative nature (Owczarek et al., 2019; Puetz and Wurm, 2019; Tripathi and Shrivastava, 2019). These can be circumvented using yeasts such as *Saccharomyces cerevisiae* and *Pichia pastoris*, which grow fast, are easy to manipulate genetically and can perform PTMs. Mammalian cells are more suitable for production of larger and more complex nanocages that might also require PTMs. The most common and used cell lines are Chinese hamster ovary (CHO), Sp2/0 and NS0. Insect cells like Sf9 can also be used for nanocage production through a baculovirus expression vector system (Owczarek et al., 2019; Puetz and Wurm, 2019; Tripathi and Shrivastava, 2019).

#### 4.2.2 Design of expression vector and preparation of recombinant host cells

The selection of host cells is accompanied by the design and construction of an expression vector/system that can drive a highly efficient nanocage expression (Puetz and Wurm, 2019). The expression vectors will comprise the gene that codes for the NVPN but also any additional genetic elements required to improve stability or enable functionalization. These can be point mutations to change or remove standard reactive residues or to introduce unnatural amino acids, or sequences of functional peptides or proteins that are fused to the N- or C-terminus or internal loops of the monomers (Diaz et al., 2018).

Most types of NVPNs reported in the literature (PepA, Dps, E2 nanocages, encapsulin, ferritin, sHSP, lumazine synthase, I3-01 nanocages, Tet8-M nanocages, TIP60 nanocages and TRAP nanocages) were produced in *E. coli* (Table 3) using pET-based expression vectors. Table 3 lists the most used key strains of *E. coli* as well as alternatives in terms of producer host organisms. Contrary to most NVPNs, vault nanocages (Figure 1A) cannot be produced in *E. coli* due to their eukaryotic origin, which implies complex post-translational modifications and protein folding (Diaz et al., 2018). In this specific case, yeast, insect, or mammalian cells must be used as producer hosts (Table 3). In yeast, the expression vector comprises a glyceraldehyde 3-phosphate dehydrogenase promoter (Wang et al., 2018) and in insect cells a Bac-to-Bac method based on infection with a recombinant baculovirus is necessary (Stephen et al., 2001; Kickhoefer et al., 2005; Poderycki et al., 2006; Champion et al., 2009; Kar et al., 2011; Kar et al., 2012; Wang et al., 2015).

**TABLE 3:** A summary of organisms and respective strains or cell lines used in the production of NVPNs.

Organism	Strain/Cell line	Protein nanocages	References
Bacteria (*E. coli*)	BL21 (DE3)	Dps	Allen et al. (2002), Suci et al. (2010)
		E2	Dalmau et al. (2009), Ren et al. (2012), Molino et al. (2013)
		Encapsulin	Moon et al. (2014)
		Ferritin	Palombarini et al. (2019), Silva et al. (2021), Wang et al. (2022)
		His6-HuHF and His6-SF nanocages	Gu et al. (2020)
		I3-01 nanocages	Hsia et al. (2016)
		Lumazine synthase	Ra et al. (2014)
		PepA	Kim et al. (2010)
		sHSP	Kim et al. (1998b), Kim R. et al. (1998), Bova et al. (2002), Flenniken et al. (2006), Choi et al. (2011)
		TIP60 nanocages	Ohara et al. (2023)
		TRAP nanocages	Malay et al. (2019), Naskalska et al. (2021)
	BL21 (DE3)B	sHSP	Flenniken et al. (2003)
	BL21 (DE3)-RIPL	I3-01 nanocages	Bruun et al. (2018)
	BL21 (DE3)C + RIL	E2	Peng and Lim (2011)
	BL21-Gold (DE3)	sHSP	Kawano et al. (2014), Guan et al. (2018), Kawano et al. (2018)
	BL21-CodonPlus (DE3)	sHSP	Murata et al. (2015)
	BL21-CodonPlus (DE3)-RIL	Ferritin	Johnson et al. (2005), Sana et al. (2010)
	JM109 (DE3)	sHSP	Wang et al. (2003)
	C43 (DE3)	Encapsulin	Cassidy-Amstutz et al. (2016)
Yeast (*P. pastoris*)	SMD1168	Vault	Wang et al. (2018)
Insect (*Spodoptera frugiperda*)	Sf9	Vault	Stephen et al. (2001), Kickhoefer et al. (2005), Poderycki et al. (2006), Champion et al. (2009), Kar et al. (2011), Kar et al. (2012), Wang et al. (2015)
Mammalian (*Homo sapiens*)	Human embryonic kidney 293F	Ferritin	Kanekiyo et al. (2013)
		Vault	Martín et al. (2022)

Once recombinant host cells with the expression vector are established, host cell banks are prepared that must be properly characterized and stored. This allows the maintenance of the reproducibility and consistency of the process, since each batch of protein nanocages will be manufactured using the same cell source (Puetz and Wurm, 2019; João et al., 2021).

#### 4.2.3 Cultivation of host cells and NVPNs expression

Following bank preparation, host cells are cultivated and the NVPN is expressed. Initially, a screening should be performed using small-scale cultures to identify and evaluate the impact of cultivation and operation parameters on protein expression levels. These parameters include media composition, temperature, agitation, aeration, cell density, pH, inducer concentration and induction time, among others (Tripathi, 2016; Tripathi and Shrivastava, 2019). After the process is defined at a small scale, scale-up and pilot studies are performed in highly controlled bioreactors as a steppingstone to industrial scale implementation. Bioreactors can be operated in batch, semi-batch, and continuous/perfusion modes, depending on the method used to supply nutrients, circulate the culture medium or recover the target NVPN. Large scale manufacturing needs to be run using optimal operation conditions that maximize expression and yield of NVPNs. Specific parameters of the bioreactor (aeration, dissolved oxygen, CO_2,_ and hydrodynamic shear) also need to be tested and optimized to guarantee high specific and volumetric productivities. A Design of Experiments (DOE) approach may be used as a more efficient strategy to optimize these parameters and understand the interaction effects between them (Tripathi, 2016; Tripathi and Shrivastava, 2019; João et al., 2021).

NVPNs studied in the literature are produced mostly in shake flask cultures with volumes up to 4 L (Zou et al., 2016a; 2016b), mainly using Luria-Bertani (LB) (Allen et al., 2002; Bellini et al., 2014; Zou et al., 2016b) or 2× YT growth media (Murata et al., 2015; Guan et al., 2018; Kawano et al., 2018; Cristie-David and Marsh, 2019). When under the control of a T7 promoter, expression is induced with the addition of 0.1 mM (Cristie-David and Marsh, 2019; Gu et al., 2020; Ohara et al., 2023) to 1 mM (Santambrogio et al., 2000; Dalmau et al., 2008; 2009; Jeon et al., 2013; Kawano et al., 2014; Zou et al., 2016a; 2016b) of isopropyl β-D-1-thiogalactopyranoside (IPTG) to the culture during the exponential growth phase. Other parameters that may vary are the induction time, between 2 h (Kim R. et al., 1998; Kim et al., 1998a; Santambrogio et al., 2000; Allen et al., 2002; Choi et al., 2011) and 30 h (Jeon et al., 2013; Zou et al., 2016a; Zou et al., 2016b; Cristie-David and Marsh, 2019; Palombarini et al., 2019), and the temperature at and after induction, which is frequently 20°C–25°C (Jeon et al., 2013; Zou et al., 2016a; Zou et al., 2016b; Palombarini et al., 2019) or 37°C (Zou et al., 2016a; Zou et al., 2016b). Overall, data regarding selection and optimization of process and operating conditions, as well as potential scale-up approaches, is very limited.

A key aspect to consider when using prokaryotic expression systems is whether protein overexpression leads to the accumulation of NVPNs as intracellular inclusion bodies. To minimize this, conditions should be optimized to increase nanocage solubility. For example, Zou and co-workers evaluated the effect of temperature (20°C, 15 h and 37°C, 5 h) on the expression of the heavy and light chains of human ferritin and on the co-expression of molecular chaperones. The authors concluded that the amount of soluble protein nanocages increased with the lower temperature and with the presence of chaperones to help in the folding process (Zou et al., 2016a; Zou et al., 2016b). If formation of inclusion bodies cannot be avoided altogether, solubilization and refolding steps must be considered in the downstream processing (Tripathi, 2016; Tripathi and Shrivastava, 2019). A study by Palombarini et al. (2019) on the expression of ferritin monomers optimized the concentration of IPTG (0.1, 0.5, and 1 mM), the induction time (4, 8, and 16 h) and the induction temperature (25°C and 37°C). The addition of 0.5 mM IPTG at 25°C for 16 h was identified as optimal and subsequently implemented at a large-scale production of ferritin nanocages by the biotechnology company GeneScript (Palombarini et al., 2019).

Martín and co-workers developed a strategy for the production of vault nanocages in mammalian cells as a faster and more efficient alternative to the traditional expression in yeast or insect cells. An engineered vault nanocage (His-tagged major vault protein) was successfully produced in the human embryonic kidney 293F cell line through transient gene expression (Martín et al., 2022).

#### 4.2.4 Alternative NVPNs expression strategy

Cell-free protein synthesis has been widely explored in recent years as an alternative to cell-based expression systems but reports of its use in NVPNs production are scarce. In one example, Mrazek described the obtention of engineered vaults using a cell-free wheat germ expression system and either a DNA vector or an mRNA encoding the major vault protein. Notably, the author was able to simultaneously package passenger molecules in the internal cavity of the formed vaults by adding them to the synthesis mixture (Mrazek, 2016). However, due to its current limitations and challenges, cell-free protein synthesis does not seem to be the most suitable strategy for consistent production of NVPNs at large-scale (Kwon and Jewett, 2015; Des Soye et al., 2018; Failmezger et al., 2018; Tran et al., 2018; Gregorio et al., 2019; Colant et al., 2021).

### 4.3 Downstream Processing

#### 4.3.1 Overview

**FIGURE 4:**
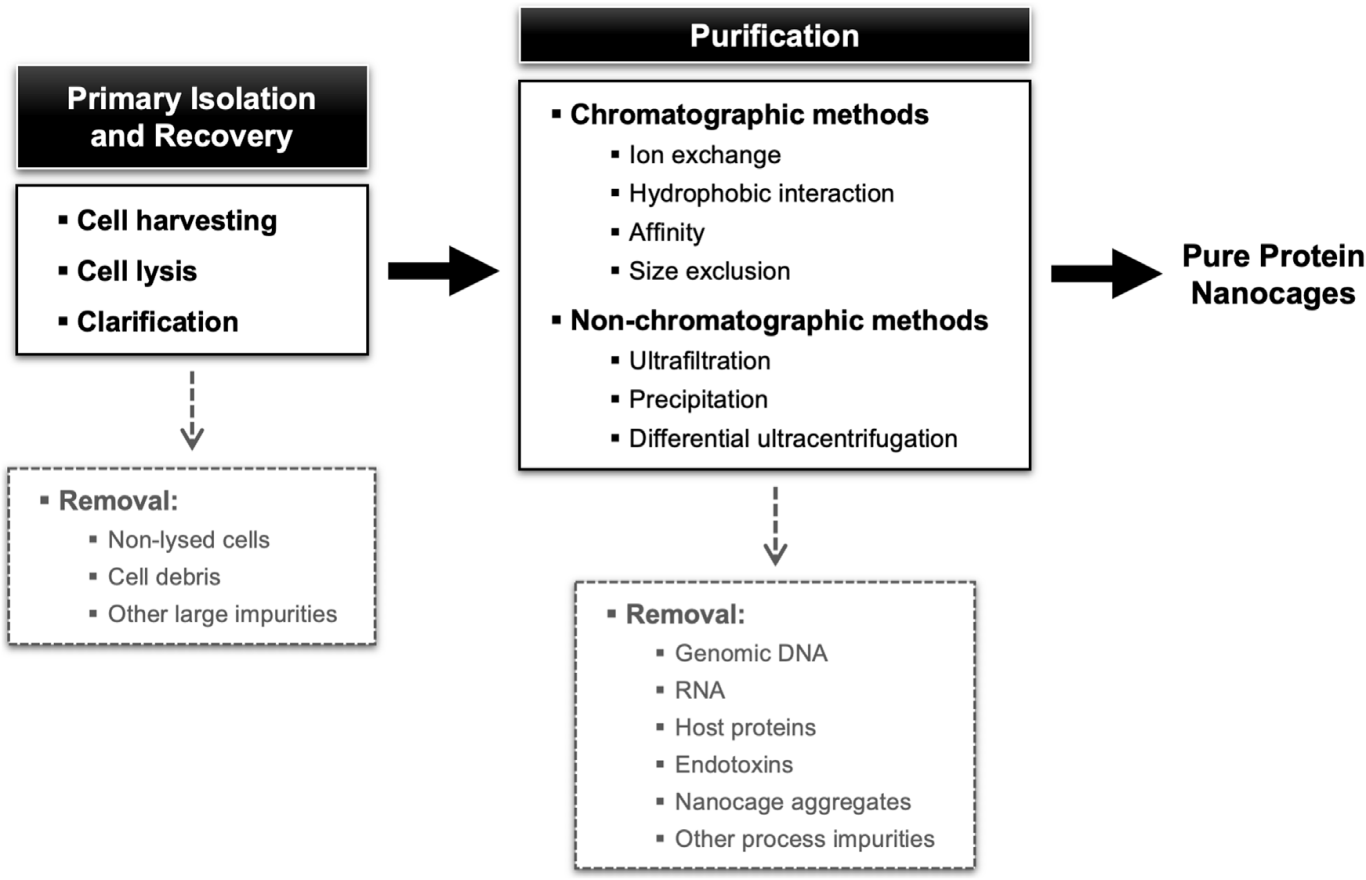
Block diagram for a standard NVPNs downstream processing showing different possibilities of unit operations.

The downstream processing encompasses the extraction, isolation, and purification of the target NVPNs from the broth culture obtained in the upstream stage. The final end-product must conform to a predetermined set of specifications that are established with the final intended use in consideration. Ideally, the overall downstream process will comprise a small number of high-yield unit operations so as to minimize complexity, residence time and processing costs (João et al., 2021). Typically, unit operations are selected and implemented that explore different physical-chemical properties of the specific protein nanocages and of the associated impurities (genomic DNA, RNA, host proteins, and endotoxins). In general, the downstream processing of NVPNs will include primary isolation and recovery steps such as cell harvesting, cell lysis and clarification, and then a number of purification steps (Tripathi, 2016; Tripathi and Shrivastava, 2019; João et al., 2021). An overall description of this downstream processing is illustrated in Figure 4.

#### 4.3.2 Primary isolation and recovery

In the first step of the downstream processing, NVPN producer cells are harvested from the culture broth, for example, by centrifugation at moderate speeds (e.g., 5,000 × *g*) (Kim et al., 1998b; Zou et al., 2016a; 2016b; Martín et al., 2022). Next, cells are resuspended in an appropriate buffer, and the intracellular nanocages are released via a specific cell lysis step. The most common options at lab scale are ultrasonication (Allen et al., 2002; Suci et al., 2010; Choi et al., 2011; Jeon et al., 2013; Zhen et al., 2013; Bellini et al., 2014; Cassidy-Amstutz et al., 2016; Martín et al., 2022), French press (Jung et al., 2002; Suci et al., 2010; Peng and Lim, 2011) and Dounce homogenization cell lysis (Stephen et al., 2001; Poderycki et al., 2006). In some instances, lysozyme is added to the lysis buffer to weaken/break down bacterial cell walls and hence improve lysis (Allen et al., 2002; Suci et al., 2010; Bellini et al., 2014). Nucleases (e.g., DNase, RNase) are also added often to lysis buffers to promote degradation of nucleic acids as they are released (Allen et al., 2002; Suci et al., 2010; Bellini et al., 2014). Protease inhibitors are also an option in some cases (Stephen et al., 2001; Poderycki et al., 2006; Bruun et al., 2018; Martín et al., 2022). For large-scale operation, high-pressure homogenizers and bead milling will certainly be more suitable than sonication, French press or Dounce homogenization cell lysis (Tripathi and Shrivastava, 2019).

After cell lysis, a clarification step based on centrifugation or filtration is normally implemented to remove cell debris and particulate matter. If a situation exists where nanocage inclusion bodies are formed, these must be separated from cell debris and recovered, solubilized and then adequately refolded (Dalmau et al., 2008; Dalmau et al., 2009; Tripathi, 2016; Tripathi and Shrivastava, 2019). For example, Jeon and co-workers have shown that inclusion bodies of ferritin nanocages can be solubilized with 8 M urea and then refolded on a nickel ion chelate affinity column with a downward gradient of urea from 8 M to 0 M (Jeon et al., 2013).

Clarified lysates containing the NVPNs are sometimes subjected to a heat treatment prior to purification. This approach explores the fact that protein nanocages are in general thermostable at high temperatures. Thus, heating clarified *E. coli* cell lysates at 65°C–90°C will promote the denaturation and precipitation of host proteins, which are subsequently removed by centrifugation (e.g., 12,000 × *g*), without affecting the structure of the thermostable nanocages (Diaz et al., 2018). This strategy was applied successfully to lysates containing a range of different nanocages, including Dps (Allen et al., 2002; Suci et al., 2010), E2 nanocages (Dalmau et al., 2008; 2009), encapsulin (Moon et al., 2014; Cassidy-Amstutz et al., 2016), ferritin (Santambrogio et al., 2000; Sana et al., 2010; Falvo et al., 2013; Zhen et al., 2013; Bellini et al., 2014; He and Marles-Wright, 2015), sHSP (Flenniken et al., 2006) and I3-01 nanocages (Hsia et al., 2016).

Several studies also report the enzymatic treatment of nanocage-containing clarified *E. coli* cell lysates. In the majority of cases, a DNase or an RNase is used directly in the clarified lysates (Santambrogio et al., 2000; Murata et al., 2015) or previously added to the lysis buffer (Allen et al., 2002; Flenniken et al., 2003; Flenniken et al., 2006; Suci et al., 2010; Bellini et al., 2014; Ohara et al., 2023) to degrade host cell nucleic acid impurities such as genomic DNA and RNA that are co-released from cells during lysis. The resulting products of degradation (e.g., short oligonucleotides) are easier to remove in subsequent purification steps. Protease inhibitors may also be added at this stage to minimize enzymatic degradation of nanocages (Bruun et al., 2018).

#### 4.3.3 Purification

The NVPNs-containing clarified lysates generated in the intermediate recovery stage and entering the final purification stage will often contain host derived impurities that must be removed to obtain a bulk product complying with final-specifications (Figure 4). Apart from genomic DNA, RNA, host proteins and other macromolecules (e.g., lipopolysaccharides in the case of *E. coli*), it is also important to remove misfolded nanocages or aggregates. Given this range of impurities, the purification stage is likely to comprise more than one step. Data on the downstream processing available in the literature primarily comes from laboratory protocols. This limited information makes it difficult to compare and weigh the merits of the different methodologies used.

##### 4.3.3.1 Chromatographic methods

Column chromatography is the preferred method for obtaining highly pure nanocages. The interaction modes explored include ion exchange (IEX), hydrophobic interaction (HIC), affinity (AC) and size exclusion (SEC). In IEX, HIC, and AC, nanocages are retained by interacting with the stationary phase, whereas impurities flowthrough and/or elute under suitable buffer conditions. Nanocages are then eluted using an adequate buffer. In general, the nanocage-containing fractions obtained are concentrated relatively to the feed (Owczarek et al., 2019; João et al., 2021). In spite of its known shortcomings, SEC is an attractive purification option because nanocages are in general larger than most host-derived impurities.

The large size of nanocages (10–100 nm) is likely to impact the performance of IEX, HIC, and AC. For once, and on account of their size, the diffusion coefficients of protein nanocages are inherently small. Furthermore, since most chromatographic matrices feature pores with diameters that seldom exceed the 30 nm (Tiainen et al., 2007), intrapore diffusion will be hindered. This may translate into internal mass transfer limitations, which can result in broad peaks, low recovery, and the need to use small flow rates. This proximity of nanocage and pore size is also likely to translate into poor binding capacities. One possible way to overcome these capacity limitations, is to use stationary phases like chromatographic membranes and monoliths that are engineered to accommodate very large pores (>200 nm). This strategy is in line with what is used for the purification of other very large biologicals such as plasmids, bacteriophages or VLPs (Prazeres, 2009; João et al., 2021).

###### 4.3.3.1.1 Ion exchange and hydrophobic interaction chromatography

In IEX, anion-exchange (AEX) resins are used almost exclusively due to the anionic nature of the outer surface of most NVPNs. However, negatively charged impurities such as nucleic acids and lipopolysaccharides can also bind to anion exchangers and thus affect binding capacity and performance. The feed to AEX columns is usually a clarified lysate of the producer host cells (microbial or mammalian). Examples of resins used include strong anion-exchangers with quaternary amine functional groups such as Uno-Q, Q Sepharose, HiTrap Q and HiPrep Q (Kim et al., 1998b; Kim R. et al., 1998; Allen et al., 2002; Dalmau et al., 2008; 2009; Choi et al., 2011; Ren et al., 2012; Kanekiyo et al., 2013; Molino et al., 2013; Kawano et al., 2014; Kawano et al., 2018; Murata et al., 2015; Peng et al., 2015) and weak anion-exchangers such as DEAE Sepharose (Gu et al., 2020). In most cases, sodium chloride gradients (up to 1 M) are employed to increase the ionic strength and elute the nanocages. If an adequate combination of stationary phase, operating conditions and elution scheme is used, a substantial amount of host impurities can be removed by AEX. HIC has also been used to purify ferritin nanocages using a Phenyl Sepharose resin (Johnson et al., 2005; Sana et al., 2010). The key disadvantage of HIC, however, is the need to use large amounts of salts to promote binding.

###### 4.3.3.1.2 Size exclusion chromatography

The removal of most impurities by AEX is usually followed by a SEC polishing step. Examples of SEC resins used here include Superose 6 (Allen et al., 2002; Dalmau et al., 2008; 2009; Kanekiyo et al., 2013; Molino et al., 2013), Superdex 200 (Choi et al., 2011; Peng et al., 2015; Gu et al., 2020), TSKgel G3000SW (Kawano et al., 2014; Kawano et al., 2018; Murata et al., 2015) and Sephacryl S-200 (Kim R. et al., 1998; Kim et al., 1998b). The key goal when using SEC for polishing is to separate nanocages from similarly sized impurities such as nanocage aggregates and misassembled variants. Baseline peak separation of the later impurities will in general be difficult to achieve due to the limitations of resolution inherent to SEC. Removal of traces of host impurities and buffer exchange are also afforded by SEC.

###### 4.3.3.1.3 Affinity chromatography

AC is often used for the purification of engineered variants of natural and artificial NVPNs. The method requires the incorporation of an affinity tag in the primary sequence of monomers, to enable the capture of assembled nanocages by an affinity resin modified with the appropriate ligand. For example, poly-histidine tags in combination with nickel ions chelate affinity resins (e.g., Ni-NTA, Ni Sepharose 6, Ni-NTA-Sefinose, HiTrap Chelating) are widely used to purify a range of protein nanocages (Kim et al., 2010; Kim et al., 2017; Jeon et al., 2013; Zou et al., 2016a; Zou et al., 2016b; Guan et al., 2018; Ohara et al., 2023). Similarly, vault nanocages produced in 293F mammalian cells were purified using commercial magnetic particles in a platform based on immobilized metal affinity chromatography (IMAC) chemistry (Martín et al., 2022). In another instance, a maltose-binding protein (MBP) domain was fused to the N-terminus of TriEst, an esterase monomer used to assemble artificial protein nanocages Tet8-M. Purification was then performed with a maltose affinity resin (MBP-Trap) (Cristie-David and Marsh, 2019). While AC provides high selectivity and specificity, leading to high purity levels, in some cases a second purification step is performed, for example, by SEC (Hsia et al., 2016; Kim et al., 2017; Bruun et al., 2018; Cristie-David and Marsh, 2019) or an AEX (Kim et al., 2010). Possible downsides of AC are related to the requirement to add affinity tags to monomers. Apart from the extra effort involved, the presence of tags can eventually compromise the self-assemble process or alter the properties of the original nanocages.

###### 4.3.3.1.4 Combinations of chromatographic steps

Although the AEX-SEC combination is widely used, Santambrogio and co-workers proposed a scheme for the purification of the heavy chain of mouse ferritin nanocages that combines a first SEC step (Sepharose 6B resin) followed by an AEX step (HiTrap Q resin) (Santambrogio et al., 2000). A similar approach was used by Jung et al. (2002) when purifying E2 nanocages (SEC with a Superdex 200 resin and AEX with a Mono-Q resin). Finally, Bova and co-workers established a three-step chromatographic purification of sHSP nanocages, which involved AEX (Mono-Q resin), HIC (Phenyl Sepharose resin) and SEC (Superose 6 resin) (Bova et al., 2002). While combinations of chromatographic steps are the norm when purifying nanocages, some reports describe the purification of NVPNs with either a single AEX (DEAE Sepharose and HiPrep Q) (Peng and Lim, 2011; Bellini et al., 2014) or a single SEC step (Superose 6, Superdex 200 and Sephacryl S-400) (Flenniken et al., 2003; Suci et al., 2010; Zhen et al., 2013; Moon et al., 2014; Wang et al., 2016; Ohara et al., 2023).

One downstream processing that is particularly interesting and unique among NVPNs involves artificial TRAP nanocages. Unlike other protein nanocages that undergo purification after self-assembly, TRAP nanocages are assembled *in vitro* in the presence of gold ions. This means that the individual sub-units of TRAP nanocages, which are composed of 11 monomeric TRAP proteins, must be purified in advance. Heddle and his group elaborated a downstream strategy that starts with a heat treatment of the clarified *E. coli* lysate containing the TRAP rings followed by AEX (Q Sepharose or HiTrap Q resins) and SEC (Superdex 200 resin) steps (Heddle et al., 2006; Heddle et al., 2007; Malay et al., 2019; Naskalska et al., 2021; Stupka et al., 2022).

###### 4.3.3.1.5 Pre-chromatography processing

In some studies the target protein nanocages are precipitated with ammonium sulphate prior to AEX or SEC chromatography to remove nucleic acid impurities (Kim R. et al., 1998; Kim et al., 1998b; Santambrogio et al., 2000; Jung et al., 2002; Hsia et al., 2016; de Turris et al., 2017; Gu et al., 2020). Differential ultracentrifugation is also described as pre-purification step before chromatography (AEX or SEC) (He and Marles-Wright, 2015). For example, encapsulins were subjected to ultracentrifugation with sucrose gradient [e.g., 10%–50% (w/v)]. However, this approach has clear drawbacks such as the need for high centrifugation speed (e.g., 100,000 × *g*) and extensive centrifugation time (e.g., 18 h), and the lack of scalability (Moon et al., 2014; Cassidy-Amstutz et al., 2016).

###### 4.3.3.1.6 Chromatographic process yields

Despite limited, some process yield data for purified NVPNs can be summarized. For example, yields of 56 mg and 25 mg per liter of cell culture were obtained for ferritin nanocages assembled from heavy chain sub-units and purified with a single AEX step (Bellini et al., 2014) and for ferritin nanocages purified with a single SEC step (Zhen et al., 2013), respectively. On the other hand, yields of 15 mg (purity >90%) and 10 mg (purity of 96%) per liter of cell culture were obtained when using Ni-affinity chromatography for the purification of the heavy and the light chains of ferritin nanocages, respectively (Zou et al., 2016a; Zou et al., 2016b). For an IMAC-based platform, Martín and co-workers verified a recovery of 90.4% in terms of the vault nanocages in the soluble fraction, obtaining a protein concentration of 20 μg mL^−1^. These authors concluded that 5 mg of the magnetic nanoparticles can capture 30 μg of vault nanocages, with a purity greater than 85% (Martín et al., 2022). A two-step chromatography purification (SEC + AEX) yielded 15 mg and 7 mg per liter of cell culture of the heavy and the light chains of ferritin, respectively (Santambrogio et al., 2000). Other reported values include 20 mg L^−1^ of cell culture for E2 nanocages purified by AEX + SEC (Dalmau et al., 2008; Dalmau et al., 2009), 400 mg L^−1^ of cell culture for artificial protein nanocages Tet8-M purified with AC (Cristie-David and Marsh, 2019) and 50 mg L^−1^ of cell culture and 40 mg L^−1^ of cell culture for encapsulin and ferritin nanocages, respectively, when purified by tandem ammonium sulphate precipitation and SEC (Cassidy-Amstutz et al., 2016; de Turris et al., 2017). A final yield of purified TRAP rings of 1–2 mg L^−1^ of cell culture was obtained with a combination of AEX and SEC steps (Heddle et al., 2006; Heddle et al., 2007; Malay et al., 2019; Naskalska et al., 2021; Stupka et al., 2022).

##### 4.3.3.2 Non-chromatographic methods

Palombarini and co-workers devised and suggested an alternative methodology for large-scale NVPNs purification that eliminates chromatographic steps while maintaining high efficiency and potentially reducing costs (Palombarini et al., 2019). Specifically, a ferritin nanocage-containing lysate obtained by sonication was subjected to an initial heat treatment and then clarified by vacuum filtration aided by diatomaceous earth. Next, the clarified lysate was purified by crossflow ultrafiltration using a 100 kDa cut-off membrane module. The system was operated in concentration and diafiltration modes, producing a stream with a final concentration of 20 g L^−1^. The process was able to eliminate critical impurities, including genomic DNA and non-targeted proteins. The methodology offers the advantage of regenerating and reusing the filtration modules multiple times, depending on the sample source and quality.

The immunogenic and pyrogenic nature of endotoxins, which mainly include lipopolysaccharides (LPS) from Gram-negative bacteria, is a significant concern when purifying biological products. Regulatory agencies such as the Food and Drug Administration (FDA) and the European Medicines Agency (EMA) establish maximum allowed limits for the quantity of endotoxins that must be met when manufacturing nanocages for biomedical applications (Schwarz et al., 2014; Franco et al., 2018). When exploring methods to remove endotoxins, it is crucial to consider the substantial size of nanocages, which makes them more prone to interact with endotoxins, as well as the requirement to preserve their architecture (Silva et al., 2021). Silva et al. (2021) employed an Endotrap HD resin in conjunction with the detergent Triton X-114 followed by a polishing step with SEC to remove endotoxins from the heavy chain of ferritin nanocages. Recovery of nanocages was 57% and the final concentration of endotoxins was 0.83 EU mL^−1^, which is lower than the maximum acceptable limit for *in vitro* and *in vivo* biomedical purposes. Additionally, Molino and his research group used a method that relied on successive washes of E2 nanocages with Triton X-114 to successfully decrease the concentration of endotoxins to acceptable values (Molino et al., 2013).

Vault nanocages whose production was performed both in *P. pastoris* SMD1168 and Sf9 insect cells were purified using a discontinuous density gradient ultracentrifugation after cell lysis (Stephen et al., 2001; Poderycki et al., 2006; Wang et al., 2018).

#### 4.3.4 Representative downstream processes

**FIGURE 5:**
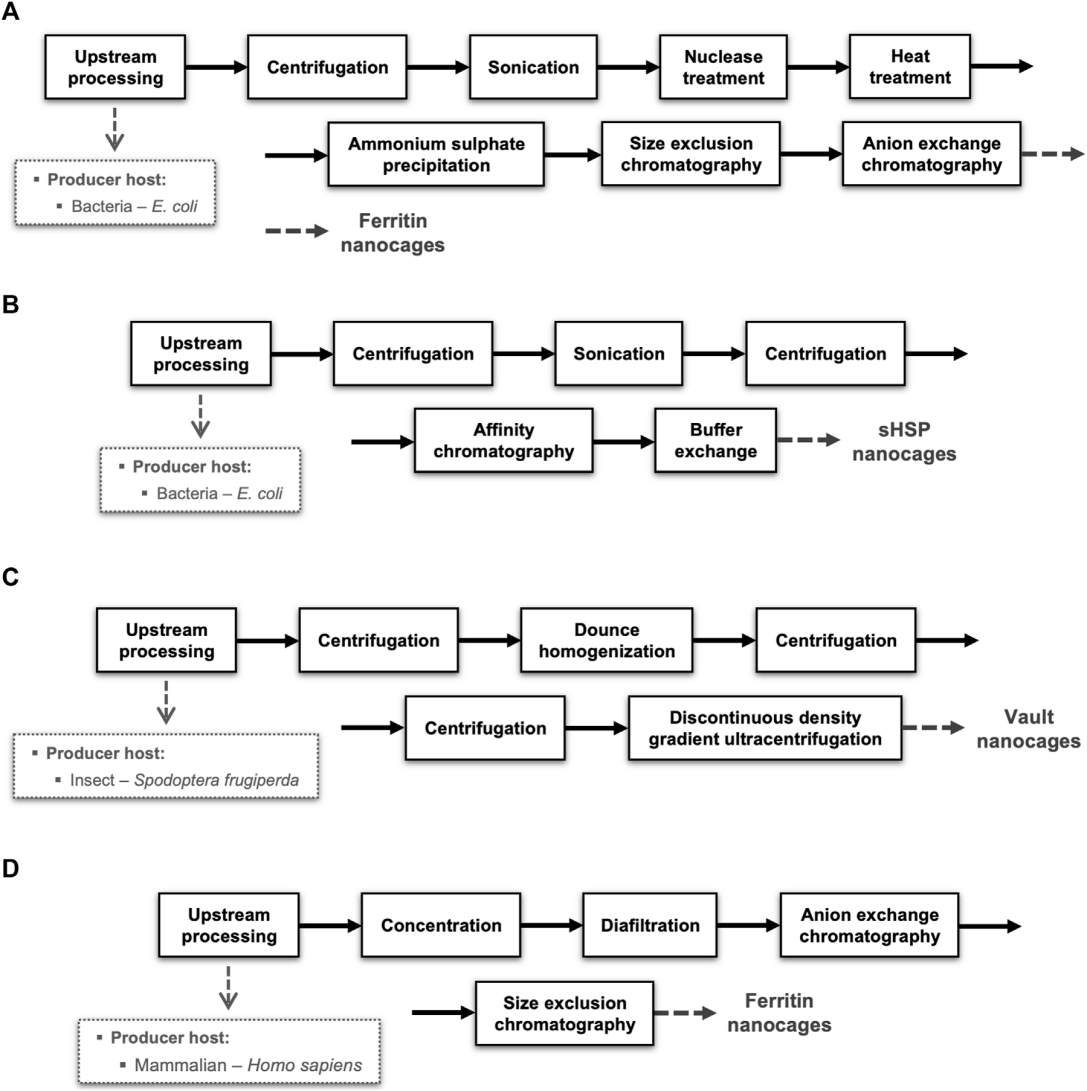
Representative examples of downstream processes implemented for distinct NVPNs produced in different organisms. Blocks diagrams **(A**,**B)** illustrate the downstream step respectively of ferritin and sHSP nanocages produced in *E. coli* and purified by chromatography. Block diagram **(C)** shows the production of vaults nanocages in insect (*Spodoptera frugiperda*) cells. Block diagram **(D)** exemplifies a downstream process of ferritin nanocages produced in mammalian (*Homo sapiens*) cells.

To illustrate the diversity of options available in terms of producer hosts and unit operations, four different block diagrams of NVPNs manufacturing processes are schematized in Figure 5. These block diagrams were adapted from the literature and are representative of the current panorama in the downstream processing of protein nanocages.

The majority of the downstream processing studies available refer to NVPNs produced in *E. coli*, which is clearly the most common producer microbial host. Blocks diagrams A (Figure 5A) and B (Figure 5B) exemplify the downstream processing respectively of ferritin (Santambrogio et al., 2000) and sHSP (Guan et al., 2018) nanocages produced in *E. coli* and purified by chromatography. On the other hand, block diagram C (Figure 5C) illustrates the production of vault nanocages in *Spodoptera frugiperda* insect cells (Stephen et al., 2001). Finally, block diagram D (Figure 5D) shows a downstream process of ferritin nanocages produced in mammalian (*Homo sapiens*) cells (Kanekiyo et al., 2013).

### 4.4 Analytical and characterization technologies

The establishment and validation of analytical and characterization technologies for assessing the structural and functional characteristics is crucial to confirm if the purified nanocage is within specifications. Furthermore, analytical techniques are also relevant to monitor the performance, robustness and consistency of the different manufacturing steps (João et al., 2021). Some well-established techniques routinely used for the determination and assessment of the biophysical characteristics of different types of NVPNs are summarized in Table 4. Dynamic light scattering (DLS) and transmission electron microscopy (TEM) of negatively stained preparations allow the estimation of the average hydrodynamic diameter of nanocages. Microscopy provides a means to observe the morphology of nanostructures and compare it with the corresponding theoretical 3D structure available in databases. Furthermore, TEM is also useful to visualize the biomineralization of nanocages. The use of analytical SEC (typical resins/columns are Superdex 200, Superose 6 and TSKgel G4000SW) enables the estimation of the molecular weight of the assembled nanostructure by reference to a calibration curve of high and low molecular weight proteins. Further, it can be used to evaluate the oligomeric state of the nanocages at the end of the bioprocess.

**TABLE 4:** A summary of well-established and less common analytical techniques used to determine and evaluate the biophysical characteristics of different types of NVPNs.

Technique		Protein nanocages	References
Standard	Dynamic light scattering (DLS)	Dps, E2, encapsulin, ferritin, sHSP and vault	Allen et al. (2002), Flenniken et al. (2003), Choi et al. (2011), Kanekiyo et al. (2013), Molino et al. (2013), Cassidy-Amstutz et al. (2016), Zou et al. (2016a), Kawano et al. (2018), Martín et al. (2022)
		I3-01 and TRAP nanocages	Hsia et al. (2016), Malay et al. (2019), Naskalska et al. (2021)
	Transmission electron microscopy (TEM)	Dps, encapsulin, ferritin, sHSP and vault	Allen et al. (2002), Flenniken et al. (2003), Choi et al. (2011), Kanekiyo et al. (2013), Cassidy-Amstutz et al. (2016), Zou et al. (2016b), Guan et al. (2018), Martín et al. (2022)
		I3-01 and TRAP nanocages	Hsia et al. (2016), Malay et al. (2019), Naskalska et al. (2021)
	Analytical SEC	Dps, encapsulin, ferritin, sHSP and vault	Kim R. et al. (1998), Kim et al. (1998b), Allen et al. (2002), Flenniken et al. (2003), Choi et al. (2011), Kanekiyo et al. (2013), Moon et al. (2014), Wang et al. (2022)
		TIP60 nanocages	Ohara et al. (2023)
Less common	High performance SEC (HP-SEC)	Ferritin	Palombarini et al. (2019), Gu et al. (2020)
	SEC with multi-angle light scattering (SEC-MALS)	TIP60 nanocages	Ohara et al. (2023)
	SEC with right-angle (RALS)/low-angle (LALS) light scattering	TRAP nanocages	Stupka et al. (2022)
	Small angle X-ray scattering (SAXS)	Ferritin	Kasyutich et al. (2010), Kim et al. (2011)
		TIP60 nanocages	Ohara et al. (2023)
	Gas-phase electrophoretic mobility molecular analyzer (GEMMA)	Vault	Poderycki et al. (2006)
	Mass spectrometry	E2	Molino et al. (2013)
		Ferritin	Kang et al. (2012), Jeon et al. (2013)
		sHSP	Flenniken et al. (2003)
	Native mass spectrometry	Encapsulin	Cassidy-Amstutz et al. (2016)
		TRAP nanocages	Malay et al. (2019)
	Electron spray mass spectrometry	Ferritin	Santambrogio et al. (2000)
	Liquid chromatography/electrospray mass spectrometry (LC/MS)	Dps	Suci et al. (2010)
		sHSP	Flenniken et al. (2006)
		TRAP nanocages	Malay et al. (2019)
	Electrospray ionization time-of-flight (ESI-TOF) mass spectrometry	Dps	Kang et al. (2008)
		Encapsulin	Moon et al. (2014)
	Matrix-assisted laser desorption/ionization time-of-flight (MALDI-TOF) mass spectrometry	E2	Dalmau et al. (2008), Dalmau et al. (2009), Peng and Lim (2011), Peng et al. (2015)
		sHSP	Kawano et al. (2014), Murata et al. (2015)
	Far-UV circular dichroism (FUV-CD) spectroscopy	E2	Jung et al. (2002), Dalmau et al. (2008), Dalmau et al. (2009), Peng and Lim (2011), Ren et al. (2011), Ren et al. (2012), Peng et al. (2015)
		Ferritin	Zou et al. (2016a)
		sHSP	Bova et al. (2002)
	Analytical ultracentrifugation	Ferritin	Kasyutich et al. (2010), Falvo et al. (2013)

Additionally, the literature reports the use of less common analytical techniques (summarized in Table 4). These techniques are used to estimate or determine structural properties of the target protein nanocages including the molecular weight, the particle size distribution, and the oligomeric state of each nanostructure. Furthermore, far-UV circular dichroism (FUV-CD) spectroscopy is used to characterize the secondary structure, the folding, and the thermostability of the protein nanocages.

Atomic force microscopy (AFM) is another promising tool for nanocage characterization. Apart from providing a means to visualize the nanostructures, AFM can be used to determine mechanical properties and study protein-protein interactions. Heddle and his group used AFM and high speed-AFM to visualize and characterize TRAP nanocages (Majsterkiewicz et al., 2022; Stupka et al., 2022). AFM was also used to analyse encapsulin nanocages (Boyton et al., 2022), ferritin nanocages (Stühn et al., 2019), lumazine synthase nanocages (Heinze et al., 2016), vault nanocages (Llauró et al., 2016) and O3-33 artificial nanocages (Heinze et al., 2016).

Although not extensively explored, methods based on fluorescence are promising due to the ease with which it is possible to label the exterior or interior of nanocages with fluorophores (e.g., Alexa Fluor 488-maleimide, Alexa Fluor 750-maleimide, fluorescein, Cys 5.5 dye) (Bova et al., 2002; Flenniken et al., 2003; 2006; Choi et al., 2011; Falvo et al., 2013; Kawano et al., 2014; Murata et al., 2015). For example, the detection and study of time-dependent fluctuations in fluorescence intensity afforded by fluorescence correlation spectroscopy (FCS) can be used to determine several physical and chemical parameters including translational and rotational diffusion coefficients (from which hydrodynamic diameters can be inferred), chemical kinetic rate constants and molecular aggregation (Schmitt et al., 2022).

Monitoring the concentration, homogeneity, and purity of the target protein nanocages throughout manufacturing is critical. Total protein is estimated by assays such as the bicinchoninic acid (BCA) (Wang et al., 2003; Wang et al., 2015; Kickhoefer et al., 2005; Heddle et al., 2006; Kar et al., 2011; Peng and Lim, 2011; Bruun et al., 2018; Malay et al., 2019; Naskalska et al., 2021; Martín et al., 2022), the Bradford (Choi et al., 2011; Zhen et al., 2013) and the biuret (Allen et al., 2002). Methods for the specific quantitation of nanocages in a mixture would be especially useful to monitor the performance of the difference steps of manufacturing. High performance liquid chromatography (HPLC) could be a potential approach, in particular based on a size exclusion chromatographic support, as it has been investigated and implemented for other large biological molecules, namely, VLPs (Effio et al., 2016; Steppert et al., 2017). SDS-PAGE is commonly used, but due to its denaturing characteristics it can only detect the presence of the nanocage monomers and other protein impurities (Flenniken et al., 2003; Kanekiyo et al., 2013; Martín et al., 2022). Native PAGE, on the other hand, can be used to check the native quaternary structure of nanocages (Santambrogio et al., 2000; Cassidy-Amstutz et al., 2016; Ohara et al., 2023).

### 4.5 Drawbacks and challenges

Developing a manufacturing process for NVPNs is a complex task, in part due to the structural and functional nature of protein nanocages. The initial challenge for process developers is to collect available data and information related to the features of the target nanocages (Moleirinho et al., 2020). Then, improving the efficiency of current processes and incorporating innovative unit operations into an integrated process is vital for improving both upstream and downstream processing of NVPNs (Moleirinho et al., 2020). The existing literature lacks data regarding the upstream processing steps, particularly concerning the optimization of the protein expression conditions and operating parameters that impact quality and concentration of protein nanocages. This optimization process is quite challenging due to the large number of involved factors and their potential interaction. To improve the purity of the final product, it is essential to establish a consistent and effective sequence of unit operations for purification. Furthermore, there should be a greater emphasis on investigating and exploring the conditions and parameters that impact purification, particularly in the chromatography stages. One example is conducting dynamic binding capacity studies. Efforts to improve the manufacturing process of NVPNs should also encompass an investment in the enhancement of current analytics and the exploration of newer techniques (Moleirinho et al., 2020). This is crucial since the analytical component of the manufacturing process is often challenging due to the complexity, time-consuming nature, or poor reliability of certain quantification methodologies.

Once a NVPNs manufacturing process is established in batch, there is potential for a shift towards continuous processing, which could bring several advantages, including high purity, increased productivity, and reduced overall process costs (Moleirinho et al., 2020; Papathanasiou and Kontoravdi, 2020). Improved analytical and monitoring methodologies that are capable of on-line and in-line analysis and can be integrated into the process will be required for continuous monitoring, process control, and product quantification (Moleirinho et al., 2020). For the upstream processing, this could entail the monitoring of standard physical and physicochemical cell culture parameters (e.g., temperature, pH, dissolved oxygen, optical density, and off-gas composition), as well as the implementation of spectroscopic (e.g., UV-visible, fluorescence, near-infrared, infrared, and Raman) and light scattering techniques [e.g., DLS and multi-angle light scattering (MALS)]. In the downstream processing, and apart from the more standard monitoring of UV, conductivity, and refractive index, light scattering sensors such as MALS should be considered (Patel et al., 2018; Aguilar et al., 2019; Tripathi and Shrivastava, 2019; Papathanasiou and Kontoravdi, 2020; João et al., 2021).

Overall, developing a robust, efficient, and scalable NVPNs manufacturing process with a reduced number of unit operations is crucial to maximize recovery yield. However, achieving this while maintaining the final quality of nanocages in a cost-effective manner remains a major challenge. As with other biological products, adjusting the manufacturing process for each type of NVPNs will be required (Moleirinho et al., 2020; João et al., 2021).

## 5 Conclusion

Protein-based nanoparticles, including natural NVPNs, have gained significant attention in bioengineering, biotechnology, and biomedicine due to their intrinsic characteristics. The design of protein-based nanocages based on natural nanostructures has also shown promise for practical applications. However, there is still a need for further research to fully understand the underlying characteristics and assembly mechanisms of nanocages. While implementing biomanufacturing processes suitable for a large-scale production is critical to bring nanocages closer to the market, few scientific studies have addressed the upstream and downstream processing of nanocages. *E. coli* is commonly used as the producer host but in the future, alternative host bacteria and other non-bacterial organisms could be explored. Further the standard production processes based on laboratory protocols should be modified and adapted for scale-up. The conceptual design of downstream processing of NVPNs is hampered by limited data and information on alternative steps and process yields. While the combination of AEX and SEC is the most used approach, complementary strategies such as aqueous two-phase extraction and crossflow ultrafiltration could be explored in the future. Finally, it is crucial to invest in more effective and simpler analytical and characterization techniques to determine and assess the structural and functional characteristics of nanocages. Clearly, further research is required to develop cost-effective NVPNs manufacturing processes.

## Figures and Tables

**Table:** 

**3D**	Three-dimensional
**AC**	Affinity chromatography
**AEX**	Anion exchange
**AFM**	Atomic force microscopy
**BCA**	Bicinchoninic acid
**BMCs**	Bacterial microcompartments
**CHO**	Chinese hamster ovary
**DEAE**	Diethylaminoethyl
**DLS**	Dynamic light scattering
**DOE**	Design of Experiments
**Dps**	DNA-binding protein from starved cells
**E2**	Dihydrolipoyl acetyltransferase
**EMA**	European Medicines Agency
**ESI-TOF**	Electrospray ionization time-of-flight
**EU**	Endotoxin Unit
**FCS**	Fluorescence correlation spectroscopy
**FDA**	Food and Drug Administration
**FUV-CD**	Far-UV circular dichroism
**GEMMA**	Gas-phase electrophoretic mobility molecular analyzer
**GVNPs**	Gas vesicle protein nanoparticles
**HIC**	Hydrophobic interaction chromatography
**HP-SEC**	High performance SEC
**HPLC**	High performance liquid chromatography
**HSP**	Heat shock protein
**IEX**	Ion exchange
**IFN-γ**	Interferon gamma
**IMAC**	Immobilized metal affinity chromatography
**IPTG**	Isopropyl β-D-1-thiogalactopyranoside
**KDPG**	2-keto-3-deoxy-6-phosphogluconate
**LALS**	Low-angle light scattering
**LB**	Luria-Bertani
**LC/MS**	Liquid chromatography/mass spectrometry
**LPS**	Lipopolysaccharides
**MALDI-TOF**	Matrix-assisted laser desorption/ionization time-of-flight
**MALS**	Multi-angle light scattering
**MBP**	Maltose-binding protein
**MJV**	Major vault protein
**MnP**	Manganese peroxidase
**mRNA**	Messenger RNA
**NTA**	Nitrilotriacetic acid
**NVPNs**	Non-viral protein nanocages
**OIPNC**	Oxygen-impermeable protein nanocages
**PAGE**	Polyacrylamide gel electrophoresis
**PDH**	Pyruvate dehydrogenase
**PepA**	Aminopeptidase
**PMBOs**	Protein membrane-based organelles
**PTMs**	Post-translational modifications
**RALS**	Right-angle light scattering
**SAXS**	Small angle X-ray scattering
**SDS-PAGE**	Sodium dodecyl-sulfate PAGE
**SEC**	Size exclusion chromatography
**sHSP**	Small HSP
**siRNA**	Small interfering RNA
**TEM**	Transmission electron microscopy
**TRAP**	trp RNA-binding attenuation protein
**VLPs**	Virus-like particles
