# Supplementary material for: Manufacturing of non-viral protein nanocages for biotechnological and biomedical applications
Source: Front Bioeng Biotechnol. 2023 Jul 13;11:1200729. doi: 10.3389/fbioe.2023.1200729 (PMC10374429; doi:10.3389/fbioe.2023.1200729)
Supplement: Supplementary file 1 [file Table1.DOCX]

Supplementary Material

Manufacturing of non-viral protein nanocages for biotechnological and biomedical applications

Jorge João, Duarte Miguel F. Prazeres^*^

*** Correspondence:** Duarte Miguel F. Prazeres: miguelprazeres@tecnico.ulisboa.pt

# Supplementary Table

**Supplementary Table S1.** Detailed list of representative studies using NVPNs in different research areas in the bioengineering, biotechnology, and biomedicine fields.

| **Research Area** | **Year** | **Protein Nanocage** | **Observations** | **References** |
| --- | --- | --- | --- | --- |
| Drug delivery | 2010 | Dps | Engineered Dps nanocages are used to deliver a photosensitizer (a compound that produces reactive oxygen species when excited by light) to biofilm microcolonies of *Aggregatibacter actinomycetemcomitans*. The original Dps from *L. innocua* were modified by functionalization with a targeting moiety (biotin) and covalent incorporation of a SnCe6 photosensitizer. The study concluded that the light-induced activity of the targeted photosensitizer reduced the viability of *A. actinomycetemcomitans* biofilm. | (Suci et al., 2010) |
|  | 2014 | Encapsulin | Encapsulins from *T. maritima* were engineered by genetic modification or chemical conjugation. Additionally, hepatocellular carcinoma cell binding peptides were displayed on the surface of the engineered encapsulins to target delivery to HepG2 cells. The anticancer prodrug aldoxorubicin was chemically bound into the nanocages. The drug was effectively delivered and released in the target cancer cells, producing a cytotoxicity effect equivalent to that obtained with free aldoxorubicin. | (Moon et al., 2014) |
|  | 2014 | Ferritin | Ferritin nanocages were used to deliver the antitumor drug doxorubicin, which exerts its cytotoxic action by targeting the nuclear DNA of human cancer cells. A genetically engineered apoferritin variant (HFn) was selected. Doxorubicin-loaded HFn nanocages showing selectivity for the target cancer cells were internalized faster and more efficiently compared to free doxorubicin, which increased the drug action efficacy. | (Bellini et al., 2014) |
|  | 2015 | sHSP | Engineered sHSP nanocages were used as a delivery platform that specifically targets human pancreatic cancer cells. A genetic engineering approach was used to coat the surface of nanocages from *M. jannaschii* with the neuropilin 1-binding peptide (iRGD), which presents affinity for the target cells. An anticancer drug, OSU03012, was successfully incorporated inside these labelled nanocages. The loaded sHSP nanocages induced the death of pancreatic cancer cells by activating the caspase cascade more efficiently when compared with the free drug. | (Murata et al., 2015) |
|  | 2018 | sHSP | Genetically modified sHSP nanocages from *M. jannaschii* were used to develop a new small interfering RNA (siRNA) delivery system. The siRNA-loaded sHSP nanocages were introduced efficiently into cancer cells and the delivered siRNA was able to significantly downregulate the desired protein expression. | (Guan et al., 2018) |
|  | 2021 | TRAP nanocages | Artificial TRAP nanocages were selected to demonstrate that NVPNs can be a valid platform for the delivery of drugs and other active compounds. Original TRAP nanocages were labelled on the surface with a cell-penetrating peptide (PTD4) and a fluorophore (Alexa-647) and the inner core was loaded with a protein cargo (a variant of a green fluorescent protein). The efficiency of the loading of the protein cargo was low, suggesting that additional future improvements are necessary. However, the labelled and loaded TRAP-nanocages successfully entered into MCF-7 and HeLa cells. | (Naskalska et al., 2021) |
|  | 2022 | Ferritin | A pH-sensitive tumor self-targeting drug delivery platform was developed based on ferritin nanocages (HFn). HFn nanocages were successfully loaded with curcumin through a disassembly/reassembly strategy. *In vitro* experiments in breast cancer cell models confirmed that the curcumin-loaded HFn nanocages have higher cytotoxicity, cellular uptake, and targeting performance. Additionally, the conjugated nanocages demonstrated superior *in vivo* therapeutic effect and lower systemic toxicity. | (Ji et al., 2022) |
| Vaccine development | 2003 | sHSP | sHSP 100 nanocages were bound to a human melanoma-associated antigen gp100 during a heat shock with the aim of developing a targeted immunotherapy. Due to its chaperone activity, it was possible to complex the sHSP 110 nanocages with the antigenic protein. These complexes were strongly immunogenic, inducing an antigen-specific IFN-γ production and a cytotoxic T-cell response. Immunization of a mice model with the complexes resulted in an antitumor response. | (Wang et al., 2003) |
|  | 2012 | Vault | The immunity produced in response to ovalbumin encapsulated in vault nanocages was characterized. The loaded nanocages induced strong anti-ovalbumin CD8+ and CD4+ T cell memory responses and a reasonable antibody production. Further, the antibody isotypes could be changed *in vivo*, which resulted in modifications of the vault nanocages. | (Kar et al., 2012) |
|  | 2013 | E2 | E2 nanocages from *B. stearothermophilus* were engineered to develop a cancer vaccine platform. The nanocages with MHC I-restricted SIINFEKL peptide epitopes displayed on the surface were loaded with dendritic cell-activating CpG molecules. The release of CpG from the E2 nanocages by a decrease in pH were able to activate bone marrow-derived dendritic cells at a lower concentration than the one needed with free CpG. The engineered and conjugated nanocages further resulted in an increased and prolonged CD8 T cell activation. | (Molino et al., 2013) |
|  | 2014 | Ferritin | Ferritin nanocages were tested to create an antigen delivery platform for dendritic cell-based vaccine development. The nanocages were engineered by adding one of two antigen peptides, SIINFEKL or ISQAVHAAHAEINEAGR, to the outer or inner surfaces. Antigen peptide-ferritin nanocages were efficiently delivered to dendritic cells, with both antigen peptides successfully inducing antigen-specific CD8+ or CD4+ T cell proliferations *in vitro* and *in vivo*. The result was a selective destruction of the antigen-specific target cells. | (Han et al., 2014) |
|  | 2014 | Lumazine synthase | An antigen delivery system to dendritic cells was investigated using lumazine synthase protein nanocages. The nanocages were externally labeled with ovalbumin peptides, SIINFEKL and ISQAVHAAHAEINEAGR, through genetic modifications. The peptides were efficient delivered by the labelled protein nanocages, followed by an efficient processing via dendritic cells *in vitro* and *in vivo*. As a consequence, proliferation of CD4+ T cells specific for both antigen peptides was induced. | (Ra et al., 2014) |
|  | 2018 | I3-01 nanocages | Artificial I3-01 protein nanocages were selected to develop a potential vaccine nanoplatform. The original nanocages were engineered to improve particle uniformity and stability, and conjugated with a SpyCatcher, allowing the binding of the antigen of interest. The modified I3-10 nanocages demonstrated a high stability to temperature, lyophilization, and freeze-thaw, among others. More importantly, they were found to have an efficiency connection of 95% to different transmission-blocking and blood-stage malaria antigens, such as the *Plasmodium falciparum* CyRPA. | (Bruun et al., 2018) |
| Bioimaging and diagnostic imaging | 2011 | sHSP | An engineered variant of sHSP nanocages from *M. jannaschii* was chemically and genetically modified by incorporating RGD and DEVD peptides and conjugating Cy5.5 and BHQ3 molecules. The modified nanocages were incorporated in cancer cells via binding and subsequent endocytic internalization. Additionally, imaging of caspase activity of the sHSP nanocages in live cells and *in vivo* experiments showed that the cages accumulate specifically in tumor tissues. The modified sHSP nanocages were used to monitor the therapeutic effect of an anticancer drug treatment by imaging their caspase activity within the tumor cells. | (Choi et al., 2011) |
|  | 2012 | Ferritin | Magnetoferritin nanoparticles were developed to target and visualize tumor tissues without the use of a contrast agent or a targeting ligand. Recombinant human heavy-chain ferritin (HFn) nanocages were loaded with iron oxide nanoparticles. The nanocages could bind to tumor cells that overexpress transferrin receptor 1. Further, the iron oxide core catalyzed the oxidation of peroxidase substrates in the presence of hydrogen peroxide, producing a color reaction that enabled the visualization of the tumor tissues. *In vivo* experiments showed that the nanocages could distinguish healthy from cancer cells with high levels of sensitivity (98%) and specificity (95%). | (Fan et al., 2012) |
|  | 2016 | Ferritin | Ferritin nanocages were used as template for the synthesis of ultrasmall copper sulfide nanoparticles inside the inner core. The metal-ferritin nanocages showed a higher photoacoustic tomography improvement for real-time *in vivo* photoacoustic imaging of tumor cells. Coupling of a copper radionuclide resulted in nanoparticles with characteristics of a good positron emission tomography imaging agent, which present higher tumor accumulation in comparison with free copper. Use of the nanocages in photothermal cancer therapy resulted in an increase of the therapeutic efficiency. Further, good biocompatibility was demonstrated both *in vitro* and *in vivo*. | (Wang et al., 2016) |
|  | 2018 | sHSP | sHSP nanocages from *M. jannaschii* were conjugated with gadolinium(III)-chelated contrast agents and iRGD peptides, which target neuropilin-1 expressed on pancreatic cancer cells. *In vitro* and *in vivo* studies showed that the molecular magnetic resonance imaging with these protein nanocages allowed detection neuropilin-1-positive cells as well as the creation of a strong signal enhancement of spontaneous pancreatic tumors in engineered mouse models. | (Kawano et al., 2018) |
| Biomineralization and nanomaterials synthesis | 2002 | Dps | Dps nanocages from *L. innocua* were used as template for the size constraining of 5 nm, homogeneous nanoparticles of the ferrimagnetic iron oxide maghemite. The electrostatic nature of the protein nanocages interior surface was found to be determinant to spatially direct the mineralization of transition metal oxyhydroxides. | (Allen et al., 2002) |
|  | 2003 | sHSP | sHSP nanocages from *M. jannaschii* were used to develop a nanoscale platform for the synthesis of inorganic materials. Two variants of the nanocages (original and engineered) were used as template to act as spatially controlled reaction vessels for iron oxide mineralization. For both variants, the result was positive, with the formation of monodispersed iron oxide nanoparticles (9 nm). | (Flenniken et al., 2003) |
|  | 2009 | sHSP | A modified variant of sHSP nanocages from *M. jannnaschii* was filled with a synthetic polymer decorated with modifiable groups. The polymer network allowed a spatial control of the reactive sites and a significant increase in the stability of the nanocages (up to 120 °C). Additionally, the introduced reactive centers were demonstrated to be functional. | (Abedin et al., 2009) |
| Biocatalysis | 2004 | Ferritin | The protein-constrained iron oxide mineral core of ferritin (ferrihydrite) was shown to be an efficient catalyst for photoreduction reactions. Specifically, ferrihydrite-mineralized ferritin nanocages catalyzed the photoreduction of Cu(II), yielding a stable and air sensitive colloidal dispersion of Cu(0). The authors show that the properties of a preformed material can be used for the specific synthesis of a second material, adjusting the desired physical properties. | (Ensign et al., 2004) |
|  | 2005 | sHSP | sHSP nanocages from *M. jannaschii* were used to create an artificial hydrogenase. Firstly, platinum nanoparticles were synthesized within the inner core of the nanocages. The platinum-sHSP nanocages (stable up to 85 °C) were able to reduce H^+^ to form H_2_ at rates comparable to the highly efficient hydrogenase enzymes and superior to those obtained with platinum nanoparticles alone. Maintenance of the integrity in the small clusters, prevention of agglomeration and control of access to active sites were the key advantages of the metallic nanocage complexes. | (Varpness et al., 2005) |
|  | 2011 | PepA | Aminopeptidase nanocages from *S. pneumonia* were used as templates for the size-controlled synthesis of ultrasmall platinum nanoparticles. The resulting bioinorganic nanohybrid catalysts were found to be active over the Glu-p-nitroanilide substrate. | (San et al., 2011) |
|  | 2015 | Vault | Manganese peroxidase (MnP) was incorporated into the vaults nanocages via fusion to a packaging domain. MnP-loaded vault nanocages were able to degrade phenol at a rate higher than the one obtained with non-encapsulated MnP. Further, the MnP conjugated to the vault nanocages displayed a significant stability in comparison with the free enzyme. | (Wang et al., 2015) |

# References

Abedin, M. J., Liepold, L., Suci, P., Young, M., and Douglas, T. (2009). Synthesis of a cross-linked branched polymer network in the interior of a protein cage. *J. Am. Chem. Soc.* 131, 4346–4354. doi: 10.1021/ja8079862.

Allen, M., Willits, D., Mosolf, J., Young, M., and Douglas, T. (2002). Protein cage constrained synthesis of ferrimagnetic iron oxide nanoparticles. *Adv. Mater.* 14, 1562–1565. doi: 10.1002/1521-4095(20021104)14:21<1562::AID-ADMA1562>3.0.CO;2-D.

Bellini, M., Mazzucchelli, S., Galbiati, E., Sommaruga, S., Fiandra, L., Truffi, M., et al. (2014). Protein nanocages for self-triggered nuclear delivery of DNA-targeted chemotherapeutics in cancer cells. *J. Control. Release* 196, 184–196. doi: 10.1016/j.jconrel.2014.10.002.

Bruun, T. U. J., Andersson, A.-M. C., Draper, S. J., and Howarth, M. (2018). Engineering a rugged nanoscaffold to enhance plug-and-display vaccination. *ACS Nano* 12, 8855–8866. doi: 10.1021/acsnano.8b02805.

Choi, S.-H., Kwon, I. C., Hwang, K. Y., Kim, I.-S., and Ahn, H. J. (2011). Small heat shock protein as a multifunctional scaffold: integrated tumor targeting and caspase imaging within a single cage. *Biomacromolecules* 12, 3099–3106. doi: 10.1021/bm200743g.

Ensign, D., Young, M., and Douglas, T. (2004). Photocatalytic synthesis of copper colloids from Cu(II) by the ferrihydrite core of ferritin. *Inorg. Chem.* 43, 3441–3446. doi: 10.1021/ic035415a.

Fan, K., Cao, C., Pan, Y., Lu, D., Yang, D., Feng, J., et al. (2012). Magnetoferritin nanoparticles for targeting and visualizing tumour tissues. *Nature Nanotech* 7, 459–464. doi: 10.1038/nnano.2012.90.

Flenniken, M. L., Willits, D. A., Brumfield, S., Young, M. J., and Douglas, T. (2003). The small heat shock protein cage from Methanococcus jannaschii is a versatile nanoscale platform for genetic and chemical modification. *Nano Lett.* 3, 1573–1576. doi: 10.1021/nl034786l.

Guan, X., Chang, Y., Sun, J., Song, J., and Xie, Y. (2018). Engineered Hsp protein nanocages for siRNA delivery. *Macromol. Biosci.* 18, 1800013. doi: 10.1002/mabi.201800013.

Han, J.-A., Kang, Y. J., Shin, C., Ra, J.-S., Shin, H.-H., Hong, S. Y., et al. (2014). Ferritin protein cage nanoparticles as versatile antigen delivery nanoplatforms for dendritic cell (DC)-based vaccine development. *Nanomedicine: Nanotechnology, Biology and Medicine* 10, 561–569. doi: 10.1016/j.nano.2013.11.003.

Ji, P., Wang, X., Yin, J., Mou, Y., Huang, H., and Ren, Z. (2022). Selective delivery of curcumin to breast cancer cells by self-targeting apoferritin nanocages with pH-responsive and low toxicity. *Drug Delivery* 29, 986–996. doi: 10.1080/10717544.2022.2056662.

Kar, U. K., Jiang, J., Champion, C. I., Salehi, S., Srivastava, M., Sharma, S., et al. (2012). Vault nanocapsules as adjuvants favor cell-mediated over antibody-mediated immune responses following immunization of mice. *PLoS ONE* 7, e38553. doi: 10.1371/journal.pone.0038553.

Kawano, T., Murata, M., Kang, J.-H., Piao, J. S., Narahara, S., Hyodo, F., et al. (2018). Ultrasensitive MRI detection of spontaneous pancreatic tumors with nanocage-based targeted contrast agent. *Biomaterials* 152, 37–46. doi: 10.1016/j.biomaterials.2017.10.029.

Molino, N. M., Anderson, A. K. L., Nelson, E. L., and Wang, S.-W. (2013). Biomimetic protein nanoparticles facilitate enhanced dendritic cell activation and cross-presentation. *ACS Nano* 7, 9743–9752. doi: 10.1021/nn403085w.

Moon, H., Lee, J., Min, J., and Kang, S. (2014). Developing genetically engineered encapsulin protein cage nanoparticles as a targeted delivery nanoplatform. *Biomacromolecules* 15, 3794–3801. doi: 10.1021/bm501066m.

Murata, M., Narahara, S., Kawano, T., Hamano, N., Piao, J. S., Kang, J.-H., et al. (2015). Design and function of engineered protein nanocages as a drug delivery system for targeting pancreatic cancer cells via neuropilin-1. *Mol. Pharmaceutics* 12, 1422–1430. doi: 10.1021/mp5007129.

Naskalska, A., Borzęcka-Solarz, K., Różycki, J., Stupka, I., Bochenek, M., Pyza, E., et al. (2021). Artificial protein cage delivers active protein cargos to the cell interior. *Biomacromolecules* 22, 4146–4154. doi: 10.1021/acs.biomac.1c00630.

Ra, J.-S., Shin, H.-H., Kang, S., and Do, Y. (2014). Lumazine synthase protein cage nanoparticles as antigen delivery nanoplatforms for dendritic cell-based vaccine development. *Clin Exp Vaccine Res* 3, 227. doi: 10.7774/cevr.2014.3.2.227.

San, B. H., Kim, S., Moh, S. H., Lee, H., Jung, D.-Y., and Kim, K. K. (2011). Platinum nanoparticles encapsulated by aminopeptidase: a multifunctional bioinorganic nanohybrid catalyst. *Angew. Chem. Int. Ed.* 50, 11924–11929. doi: 10.1002/anie.201101833.

Suci, P., Kang, S., Gmür, R., Douglas, T., and Young, M. (2010). Targeted delivery of a photosensitizer to Aggregatibacter actinomycetemcomitans biofilm. *Antimicrob Agents Chemother* 54, 2489–2496. doi: 10.1128/AAC.00059-10.

Varpness, Z., Peters, J. W., Young, M., and Douglas, T. (2005). Biomimetic synthesis of a H2 catalyst using a protein cage architecture. *Nano Lett.* 5, 2306–2309. doi: 10.1021/nl0517619.

Wang, M., Abad, D., Kickhoefer, V. A., Rome, L. H., and Mahendra, S. (2015). Vault nanoparticles packaged with enzymes as an efficient pollutant biodegradation technology. *ACS Nano* 9, 10931–10940. doi: 10.1021/acsnano.5b04073.

Wang, X.-Y., Chen, X., Manjili, M. H., Repasky, E., Henderson, R., and Subjeck, J. R. (2003). Targeted immunotherapy using reconstituted chaperone complexes of heat shock protein 110 and melanoma-associated antigen gp100. *Cancer Res* 63, 2553–2560.

Wang, Z., Huang, P., Jacobson, O., Wang, Z., Liu, Y., Lin, L., et al. (2016). Biomineralization-inspired synthesis of copper sulfide–ferritin nanocages as cancer theranostics. *ACS Nano* 10, 3453–3460. doi: 10.1021/acsnano.5b07521.
